# In Silico Design and Evaluation of TcGAPDH as a Vaccine Against Chagas Disease: A Reverse Vaccinology Approach

**DOI:** 10.1007/s11686-026-01221-4

**Published:** 2026-02-16

**Authors:** Veronica Nava-Cuamatzi, Ricardo Enrique Buendia-Corona, María Cristina González-Vázquez, Maria Lilia Cedillo-Ramirez, Alejandro Carabarin-Lima

**Affiliations:** 1 Posgrado en Microbiología, Centro de Investigaciones en Ciencias Microbiológicas. Instituto de Ciencias, Benemérita Universidad Autónoma de Puebla. Ciudad Universitaria, San Manuel, 72570 Puebla, Mexico; 2 Departamento de Ciencias Quimico-Biológicas, Universidad de Las Americas Puebla, Ex Hacienda Sta. Catarina Martir S/N, San Andres Cholula, 72810 Puebla, Mexico; 3 Facultad de Ciencias Biológicas, Benemérita Universidad Autónoma de Puebla, Ciudad Universitaria, San Manuel, 72570 Puebla, Mexico

**Keywords:** *Trypanosoma cruzi*, Chagas disease, TcGAPDH, Reverse vaccinology, Vaccine, Immunoinformatics, Toll-like receptor interactions

## Abstract

Purpose

The current treatment for Chagas disease, caused by *Trypanosoma cruzi*, presents significant limitations due to its variable efficacy depending on the stage of the disease and its associated adverse effects. This issue has driven research and the development of new therapeutic and preventive strategies, with a particular emphasis on vaccine design.

Materials and methods

In the present study, a bioinformatics approach was employed to evaluate *T. cruzi* glyceraldehyde-3-phosphate dehydrogenase (TcGAPDH) as a potential vaccine candidate by analysing its physicochemical and immunogenic properties.

Results

In this analysis, epitopes for B and T lymphocytes were predicted, and a predictive immune response determined that TcGAPDH has the capacity to induce a Th1-type response driven by IFN-γ and IL-2. Finally, molecular docking and dynamics simulation demonstrated favourable interaction between TcGAPDH and TLR2/TLR4 receptors, which remained constant throughout a 300 ns simulation.

Conclusions

These findings highlight the immunogenic potential of TcGAPDH and support its feasibility as a promising candidate for the development of a vaccine against Chagas disease.

**Supplementary Information:**

The online version contains supplementary material available at 10.1007/s11686-026-01221-4.

## Introduction

Chagas disease (CD) is classified as one of the 20 diseases that comprise the group of neglected tropical diseases (NTDs), receiving disproportionately low attention and funding relative to its disease burden, with severe health, social, and economic consequences for affected populations [1, 2]. This disease is a parasitic infection caused by *Trypanosoma cruzi*, which is transmitted mainly through contact with the faeces or urine of vector insects known as kissing bugs that belong to the genus Triatominae [3].

Although it is primarily found in endemic areas of 21 Latin American countries, where it poses a significant public health issue, it has become one of the most pressing emerging health problems in Europe and the United States due to the migratory movements of individuals from endemic areas [4, 5]. According to the World Health Organization (WHO, 2024), an estimated 6–7 million people worldwide are currently infected with *T. cruzi*, with approximately 75 million people at risk of infection in endemic areas. The annual incidence is estimated at 30,000–40,000 new cases, resulting in approximately 12,000 deaths per year [2, 6].

*T. cruzi* belongs to a heterogeneous species consisting of strains or isolates circulating between mammalian hosts and insect vectors; this heterogeneity has been extensively studied by biological, biochemical and molecular methods [7]. Currently, *T. cruzi* isolates have been classified into seven discrete typing units (DTUs), named TcI to TcVI and Tcbat. These DTUs exhibit distinct genotypes and phenotypes, as well as evolutionary relationships, ecological, and epidemiological associations that influence aspects such as pathogenesis, tropism, and drug resistance. However, a definitive correlation between disease severity, treatment efficacy and parasite lineage has not been established [8, 9].

Chagas disease has two phases: acute and chronic. In the acute phase, 5–10% of symptomatic cases may develop serious complications such as myocarditis or meningoencephalitis, which can be fatal in some cases. In the chronic phase, 30–40% of infected individuals develop symptoms 10 to 30 years after infection. These manifestations include cardiac (cardiopathies) and/or gastrointestinal (megaesophagus and megacolon) conditions, which are the leading cause of mortality in this disease [7, 10].

Diagnosis of Chagas disease is essential for the reporting of new cases. However, during the acute phase, it is difficult due to heterogeneous and non-specific clinical findings, which limit timely treatment [11].

Currently, only two drugs are approved for the treatment of this disease, nifurtimox and benznidazole, both with trypanocidal activity against all parasitic forms. In the acute phase, the use of these drugs has demonstrated a curative efficacy of 50% to 80%, whereas in the chronic phase, their effectiveness is significantly reduced, reaching only 8% to 20% [12, 13]. Despite their proven efficacy, especially in the acute phase, the use of both drugs is limited by their high toxicity, which can cause renal and hepatic dysfunction, adverse effects on the skin and gastrointestinal tract, and harm during pregnancy. For this reason, the development of new, more effective and safer therapeutic and preventive methods has become a priority research objective [14, 15].

In recent years, several vaccines have been studied and developed as part of an approach for new preventive methods. However, to date, none have reached the clinical trial stage. Some of the strategies used for vaccine development include immunizations with killed or attenuated parasites, cell fractionation, purified or recombinant proteins, or with DNA [16]. Reverse vaccinology has also emerged as a promising approach to facilitate the identification of proteins with immunogenic potential for the development of vaccines against human pathogens [12].

Glyceraldehyde-3-phosphate dehydrogenase (GAPDH; EC 1.2.1.12) is primarily described as a key enzyme in the glycolytic pathway, where it catalyses the reversible oxidative phosphorylation of glyceraldehyde-3-phosphate (G3P) to 1,3-bisphosphoglycerate (BPG) in the presence of NAD + and inorganic phosphate [17]. Recent studies have shown that GAPDH is a moonlighting protein. In addition to its primary role in energy metabolism, GAPDH is involved in various independent functions, including DNA repair, regulation of gene expression, cell signalling, membrane fusion and transport, as well as interactions with RNA and other proteins. It has been reported that the properties that enable GAPDH to perform these additional functions are regulated by its oligomerization state, post-translational modifications, and cellular localization [18, 19].

In various microbial species, GAPDH is localized on the cell surface, enabling it to interact with host proteins, such as serum proteins, extracellular matrix components, and cytoskeletal proteins. These interactions support their role as a ligand for the recognition and invasion of host cells [20]. In bacterial pathogens including *Streptococcus pyogenes* and enteropathogenic *Escherichia coli*, surface-exposed GAPDH has a role as an adhesin by binding host extracellular matrix proteins including fibronectin, laminin, and plasminogen, thereby promoting host-tissue adhesion, proteolytic remodelling of the extracellular matrix, and enhanced invasiveness [21, 22].

The GAPDH secreted by pathogens may function as a virulence-associated immunomodulatory antigen to induce both innate and adaptive immunity [20]. Immunisation studies across phylogenetically diverse pathogens have consistently demonstrated robust immunogenic properties. In bacterial systems, GAPDH from *Listeria monocytogenes* generates peptide-restricted antibodies in the absence of adjuvants and induces specific T cell responses with efficiencies comparable to the well-characterised immunodominant antigen listeriolysin O [23]. Similarly, immunisation of mice with recombinant GAPDH from Group B *Streptococcus* (GBS) elicits high-titre IgG antibodies that, notably, do not cross-react with human GAPDH, and maternal vaccination provides protection to offspring against lethal infection by blocking IL-10 production and restoring neutrophil recruitment to infected tissues [24, 25]. In parasitic systems, GAPDH vaccination induces both humoral and cell-mediated immune responses. DNA vaccination with GAPDH from *Eimeria* species significantly increases proportions of CD4⁺ and CD8⁺ T lymphocytes whilst upregulating expression of IFN-γ, IL-2, IL-4, IL-17, and TGF-β, conferring protection against experimental challenge with multiple *Eimeria* species [26]. For *Schistosoma mansoni*, GAPDH immunisation predominantly elicits a Th1-biased response characterised by elevated IFN-γ and IL-2 production along with IgG2a and IgG2b antibody isotypes, resulting in significant reductions in worm burden [27, 28]. These collective findings demonstrate that GAPDH, despite its housekeeping function, serves as a broadly immunogenic antigen capable of eliciting protective Th1-predominant responses with both antibody and T cell components across phylogenetically distant organisms.

Based on this evidence, TcGAPDH was selected for immunoinformatic evaluation as a vaccine candidate against Chagas disease. As a conserved metabolic enzyme, TcGAPDH is expected to exhibit minimal sequence variation across different *T. cruzi* discrete typing units (DTUs) and developmental stages, potentially offering broader vaccine coverage compared to the highly polymorphic trans-sialidase and mucin families that show extensive strain variability and are located in the disruptive genomic compartment [29].

In this study, multiple bioinformatic tools were used to evaluate the physicochemical and immunogenic properties of *T. cruzi* glyceraldehyde-3-phosphate dehydrogenase (TcGAPDH). These analyses allowed the prediction of antigenic and non-allergenic properties of the protein, as well as the identification of epitopes recognised by MHC-I, MHC-II molecules, and B cells. Furthermore, immune response simulation predicts that TcGAPDH could induce a Th1-type response, demonstrated by increased production of IgM, IgG, IFN-γ, IL-12, and IL-2. Additionally, molecular docking and dynamics simulations showed an affinity of TcGAPDH for TLR-2 and TLR-4 receptors. These results indicate that TcGAPDH could activate immediate immune responses, supporting its potential as a vaccine candidate.

## Materials and Methods

### Retrieval and Analysis of *Trypanosoma cruzi* Glyceraldehyde-3-Phosphate Dehydrogenase (TcGAPDH) Protein Sequences

The TcGAPDH protein sequences used for this study were obtained from TriTrypDB database release [30, 31]. The selection of sequence data was based on the criteria that the protein-coding genes were complete. In total, 10 sequences corresponding to strains belonging to DTU I, II, and VI were retrieved. The amino acid sequences were aligned in Clustal Omega [32, 33]. After multiple alignment analysis, a reference sequence was obtained.

### Physicochemical Characterization, Antigenicity and Allergenicity Assessment, and Homology Analysis with Human GAPDH

The physicochemical properties and safety of the TcGAPDH protein were determined in silico using the following tools: ProtParam from ExPASy [34, 35]; VaxiJen v2.0, with a threshold of 0.4 and the parasite target model, employing the alignment-independent approach based on auto cross-covariance (ACC) transformation of protein sequences [36, 37]; and AllerTOP v2.1, which applies the ACC transformation method with default parameters to provide a binary classification (allergen/non-allergen) based on its internal training dataset [38, 39]. To determine homology between TcGAPDH and human GAPDH (GenBank ID: P04406), both amino acid sequences were aligned using Clustal Omega with default parameters.

### Immunogenic Potential Prediction

The immunogenic potential of the TcGAPDH protein was predicted using tools designed to identify Major Histocompatibility Complex (MHC-I and MHC-II) epitopes that target CD8 + and CD4 + T cells, as well as B cell-specific epitopes. The tools used included the Immune Epitope Database & Tools (IEDB) from the La Jolla Institute, employing NetMHCpan 4.1 EL for MHC-I epitopes and NetMHCIIpan 4.1 EL for MHC-II epitopes (both recommended epitope predictors-2023.09), with a percentile rank threshold of ≤ 10 for epitope selection [40, 41]; the NetCTL-1.2 Server for MHC-I cross-validation, considering 9-mer peptides with an epitope threshold of ≥ 0.75, using default weights for C-terminal cleavage (0.15) and TAP transport efficiency (0.05) [42, 43]; ProPred for MHC-II epitope cross-validation, which predicts 9-amino acid core regions within MHC-II binding peptides using neural network algorithms, with a threshold of 5% (only the 15-amino acid epitopes determined by IEDB are reported in this study, while ProPred cores were used exclusively for validation purposes) [44, 45] BepiPred-2.0 for B-cell epitope prediction, with a threshold of ≥ 0.5 [46, 47] and ABCPred for B-cell epitope cross-validation, using artificial neural networks with a window length of 16 amino acids and threshold of ≥ 0.51(only the B-cell epitopes selected from BepiPred-2.0 are reported in this study, while ABCPred predictions were used exclusively for validation purposes) [48, 49].

The selection of MHC-I and MHC-II epitopes was based on their representativeness in reference Human Leukocyte Antigen (HLA) supertypes, mainly associated with the Latin American population [50–52]. The specific alleles used for epitope prediction are detailed in Tables 4 and 5. Additionally, for MHC-I epitopes, the presence of proteasomal cleavage sites was evaluated using the NetChop 3.1 server, with the C-term 3.0 method and a threshold of 0.5 [53, 54].

### Immune Response Simulation

The stimulated immune response by the immunization of the TcGAPDH protein was predicted using the C-ImmSim server [55, 56]. This platform enables the assessment of global immunogenicity profiles for protein based on their amino acid sequence. Two simulation scenarios were performed: a negative control with a short non-immunogenic peptide (GGGGGG) and an immunization protocol with TcGAPDH protein, both administered with the generic adjuvant included in the program. Both simulations modelled a three-dose immunization protocol, with injections administered on days 1, 7, and 14. Default parameters were used, including 300 simulation time steps (equivalent to 100 days), injection volume of 1,000 units, and adjuvant concentration of 100 units , to evaluate the immune system's response under a prime-boost immunization scheme.

### Refinement and Assessment of Crystallized Structures for Molecular Docking and Molecular Dynamics Simulations

For molecular docking and molecular dynamics simulations, the following protein structures for TcGAPDH, TLR-2 and TLR-4 were retrieved from the Protein Data Bank (PDB) [57, 58] with IDs: 3IDS, 2Z7X and 4G8A, respectively. We use reference structures as controls for all measured structural properties. The reference structure of TLR2 in complex with the SSL3 protein (PDB ID: 5D3I) [59]and the reference structure of TLR4 in complex with the MD-2 protein (PDB ID: 4G8A) [60].

Each protein complex obtained from the PDB was subjected to structural preprocessing involving the removal of co-crystallized ligands, water molecules, and solvent components to isolate the desired protein structure. This purification step was performed using UCSF Chimera software (version 1.18) [61] to prepare standardized structures for subsequent computational analysis.

To reduce steric clashes between residues' side chains, all protein structures were minimized at 500 and 1000 steps using the steepest descent gradient with FF14SB force field [62] in UCSF Chimera 1.18. Clash score reduction was measured with the Molprobity platform [63, 64].

### Molecular Docking Simulations

Molecular docking simulations were employed to determine the three-dimensional structural conformations of ligand-receptor complexes, characterize putative binding sites, and evaluate binding affinities between the TcGAPDH protein and the membrane-associated Toll-like receptors TLR-2 and TLR-4. The docking simulations were performed using the HDOCK server [65, 66], with default parameters, which generate more than 100 predictions ranked according to docking scores. Due to the high variability characteristic of protein–protein docking simulations, visual inspection of the top 10 scoring conformations was performed, and hydrogen bond interactions were assessed using the Pictorial database of 3D structures in the Protein Data Bank (PDBsum) [67, 68]. Based on conformational similarity to reference controls and favourable hydrogen bond interactions, a complex was selected for subsequent computational analyses.

### Molecular Dynamics Simulations

To explore the mechanistic binding properties and molecular interactions between the TcGAPDH protein and TLR-2 & TLR-4 receptors, molecular dynamics simulations were performed on the selected complex. Parametrizations of docking complexes were constructed using the Tleap module from the AmberTools24 software package [69, 70]. The FF19SB force field [62] and the OPC water model [71] were employed for all complexes. Each system was solvated in a cubic water box with a minimum distance of 8 Å between the solute surface and box boundaries. Na + and Cl- ions were added for charge neutralization. Energy minimization was performed using the steepest descent algorithm for 30,000 steps to optimize the organization of water molecules and remove steric clashes. Before production simulations, the systems underwent two ns of gradual heating from 0 to 300 K under the NVT ensemble, with harmonic restraints (10 kcal/mol·Å^2^) applied to solute heavy atoms to prevent structural distortion. Temperature was controlled using the Langevin thermostat with a collision frequency of 2 ps⁻^1^. This was followed by two ns of pressure equilibration at 1 atm under the NPT ensemble, employing the Berendsen barostat with a pressure relaxation time of 2 ps, during which restraints were gradually released. Subsequently, 300 ns molecular dynamic production simulations were conducted under the NPT ensemble at 300 K and 1 atm for all complexes, including control systems using the pmemd module from AmberTools24 accelerated by cuda libraries [72]. A timestep of 2 fs was employed, with covalent bonds involving hydrogen atoms constrained using the SHAKE algorithm.

To assess the structural behaviour and conformational dynamics of the system, and to evaluate the persistence of intermolecular interactions within the complex throughout the simulation period, molecular dynamics trajectories were analysed using the cpptraj module [73] of the AmberTools24 software package. Root mean square deviation (RMSD) values were calculated to evaluate the structural motion of both the ligand and receptor over the simulation period, compared to their initial positions as a reference. Interactions by hydrogen bonds were analysed, with both ligand and receptor serving as hydrogen bond donors and acceptors. Additionally, native and non-native contacts between ligand and receptor with 3.5 Å distance as a cutoff were evaluated.

Binding free energies between ligand and receptor complexes were calculated using the Molecular Mechanics/Generalized Born Surface Area (MM/GBSA) approach, as implemented in MMPBSA.py [74], with structural sampling conducted at 10-frame intervals.

Three-dimensional structural representations of the ligand-receptor complexes were generated and visualized using ChimeraX software [75]. Molecular dynamics trajectories were analysed graphically and represented using GraphPad Prism statistical software [76]. Rendering and animation of simulation data were conducted using the VMD software package [77].

## Results

### *Trypanosoma Cruzi* Glyceraldehyde-3-Phosphate Dehydrogenase and a Reference Sequence

A total of 10 glyceraldehyde-3-phosphate dehydrogenase (TcGAPDH) coding sequences were recovered from the TriTrypDB database, corresponding to 10 isolates classified as DTU's I, II and VI (Table 1).

**Table 1:** *T. cruzi* isolates with the complete glyceraldehyde-3-phosphate dehydrogenase (TcGAPDH) coding sequence identified in TriTrypDB

DTU	*T. cruzi* isolate	TriTrypDB access code
I	Dm28c	BCY84_01123_t1
	Sylvio X10/1	TCSYLVIO_003325-t26_1
	G	TcG_01020_t1
	Brazil A4	TcBrA4_0027980-RA
II	Berenice	ECC02_000028_t1
	YC6	TcYC6_0098230-RA
VI	CL	TcCL_ESM01054_t1
	TCC	C3747_28g40-t42_1
	CL Brener Non-Esmeraldo-like	TcCLB.509065.60
	CL Brener Esmeraldo-like	TcBEL_TcCLB.506943.50

The GAPDH gene coding sequences identified were obtained and analysed in both the nucleotide and amino acid sequence levels. Multiple sequence alignment using ClustalW demonstrated that all sequences comprised 359 amino acids (1,080 nucleotides). At the amino acid level, most of the sequences presented an identity percentage ≥ 99.44%. Overall, two constant mutations were identified among the sequences. The first was located at position 62, where four strains presented a glutamic acid (E), five a threonine (T) and one an asparagine (N). The second mutation was located at position 358, where one strain showed a lysine (K) and the rest an arginine (R).

**Fig. 1:**
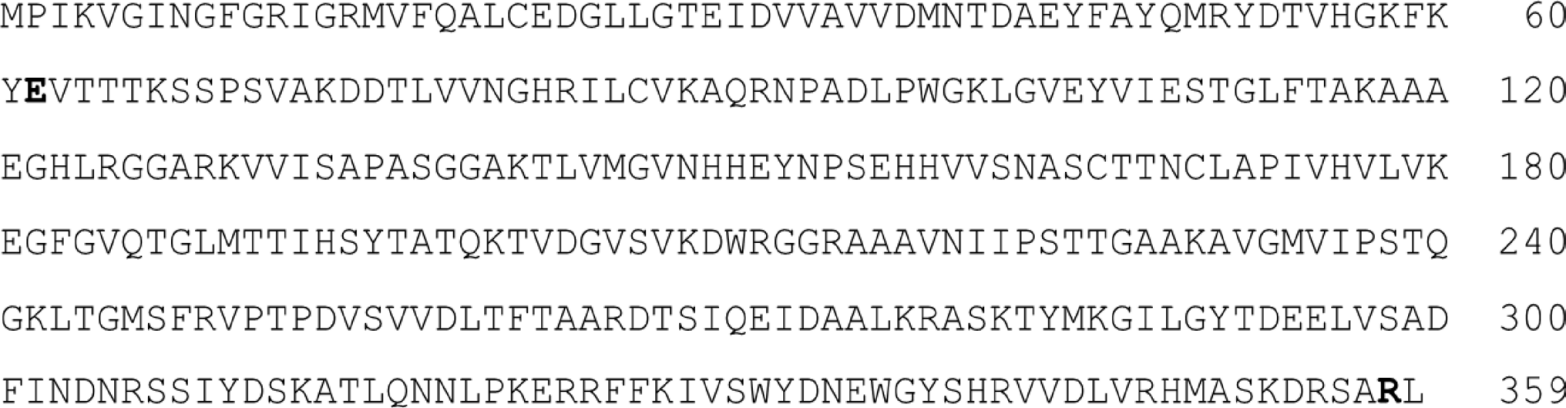
Representative *Trypanosoma cruzi* glyceraldehyde-3-phosphate dehydrogenase (TcGAPDH: BCY84_01123_t1) amino acid sequence (359 aa) corresponding to DTU-I. Bold letters denote mutations T62E-N62E and K358R consistently found in DTU II and VI relative to DTU I sequences

Due to the high percentage of amino acid sequence identity among the strains, the use of a single representative sequence was considered for further analysis. A sequence corresponding to DTU-I (Dm28c: BCY84_01123_t1), a widely distributed DTU across the Americas [9], was selected as the reference (Fig. 1).

### Physicochemical, Antigenicity and Allergenicity Property Evaluation

The physicochemical properties of the TcGAPDH protein were determined through ProtParam analysis. This computational assessment provided critical parameters, including molecular weight, predicted half-life in different biological systems, and stability measures (Table 2). The analysis revealed a molecular weight of 39,060.55 Da and an instability index of 26.74, indicating stable protein properties. The GRAVY index of -0.147 demonstrated the hydrophilic properties of the protein. Additionally, the analysis predicted extended half-life values across different organisms, further supporting the stability characteristics of this protein.

**Table 2:** TcGAPDH physicochemical properties obtained with ProtParam

Parameter	Obtained values
Molecular weight	39,060.55 Da
Theoretical pI	8.87
Formula	C_1725_H_2744_N_486_O_520_S_14_
Estimated half-life hours: mammals and in vitro, yeast and in vivo*,* and bacteria, respectively	> 30, > 20, y > 10
Instability index (II)	26.74
Aliphatic index	84.15
Grand average of hydropathicity (GRAVY)	-0.15

In silico safety profiling was conducted to assess the therapeutic potential of the TcGAPDH protein. VaxiJen v2.0 and AllerTOPv2.1 servers were utilized for antigenicity and allergenicity predictions, respectively. As presented in Table 3, the analysis demonstrated that the TcGAPDH protein exhibits antigenic potential with non-allergenic properties, suggesting its ability to generate immune responses without inducing adverse allergic reactions. Additionally, a homology analysis was conducted through sequence alignment to assess potential cross-reactivity between the protein candidate and host proteins, thereby minimizing the risk of autoimmune responses. The alignment analysis revealed a sequence identity of 53.29% between TcGAPDH and human GAPDH (Figure S1), a level of conservation that will require careful evaluation of epitope-level cross-reactivity in future experimental studies to assess potential autoimmunity risks.

**Table 3:** TcGAPDH safety properties obtained with VaxiJen v2.0 and AllerTOP v2.1

Properties	
Antigenicity	Antigen
Allergenicity	Non-allergen

### MHC-I, MHC-II, and B Cell Epitopes Prediction

In silico epitope prediction was performed to assess the immunogenic potential of the TcGAPDH protein. The analysis identified seven epitopes with predicted binding affinity to predominant MHC-I alleles in the Latin American population (Table 4). Proteasomal cleavage site prediction revealed heterogeneous processing patterns: two epitopes contained five predicted cleavage sites; one epitope contained three sites; three epitopes contained two sites each one; and one epitope contained a single cleavage site.

**Table 4:** Predicted MHC-I epitopes of the TcGAPDH protein using IEDB and NetCTL1.2

MHC-I sequences	Amino acid position	Proteasomal cleavage sites	HLA supertypes
RILCVKAQR	84–92	5	A31, A33, A68, A11, A30, B57
GLMTTIHSY	188–196	2	A31, A32, A11, A01, B57, A68
KTVDGVSVK	201–209	3	A31, A11, A68, B57
MVIPSTQGK	234–242	2	A31, A68, A11, A33, B58
ATLQNNLPK	314–322	1	A31, B57, A68
RFFKIVSWY	325–333	2	A31, A30, B57, A33
DLVRHMASK	345–353	5	A31, A11, A68

**Fig. 2:**
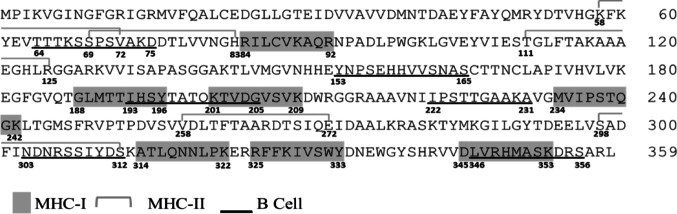
Representative *Trypanosoma cruzi* glyceraldehyde-3-phosphate dehydrogenase (TcGAPDH) with predicted epitopes highlighted. MHC-I epitopes are shaded in grey, MHC-II epitopes are indicated with upper brackets, and B-cell epitopes are underlined

Additionally, in silico analysis predicted five MHC-II epitopes (Table 5) with high binding affinity to prevalent allelic variants in Latin American populations, and six linear B-cell epitopes were also predicted (Table 6). Notably, the analysis revealed that several B-cell epitopes demonstrated overlapping sequence regions with both MHC class I and MHC class II epitopes within the protein sequence (Fig. 2).

**Table 5:** Predicted MHC-II epitopes of the TcGAPDH protein using IEDB

MHC-II sequences	Amino acid position	HLA supertypes
SPSVAKDDTLVVNGH	69–83	DRB1, DRB3, DQA1, DQB1, DPA1
SADFINDNRSSIYDS	298–312	DRB3, DRB1, DPA1, DPB1, DRB5
TGLFTAKAAAEGHLR	111–125	DRB1, DQA1, DQB1, DPA1, DRB5, DRB3
KFKYEVTTTKSSPSV	58–72	DRB1, DRB5, DRB3
VDLTFTAARDTSIQE	258–272	DRB1, DQA1, DQB1, DRB3

**Table 6:** Predicted B-cell epitopes of the TcGAPDH protein using BepiPred-2.0

Sequences	Amino acid position	Overlapping with predicted HLA epitopes
TTTKSSPSVAKD	64–75	Yes (2)
YNPSEHHVVSNAS	153–165	No
IHSYTATQKTVDG	193–205	Yes (2)
IPSTTGAAKA	222–231	No
NDNRSSIYDS	303–312	Yes (1)
LVRHMASKDRS	346–356	Yes (1)

### Immune Response Simulation

**Fig. 3:**
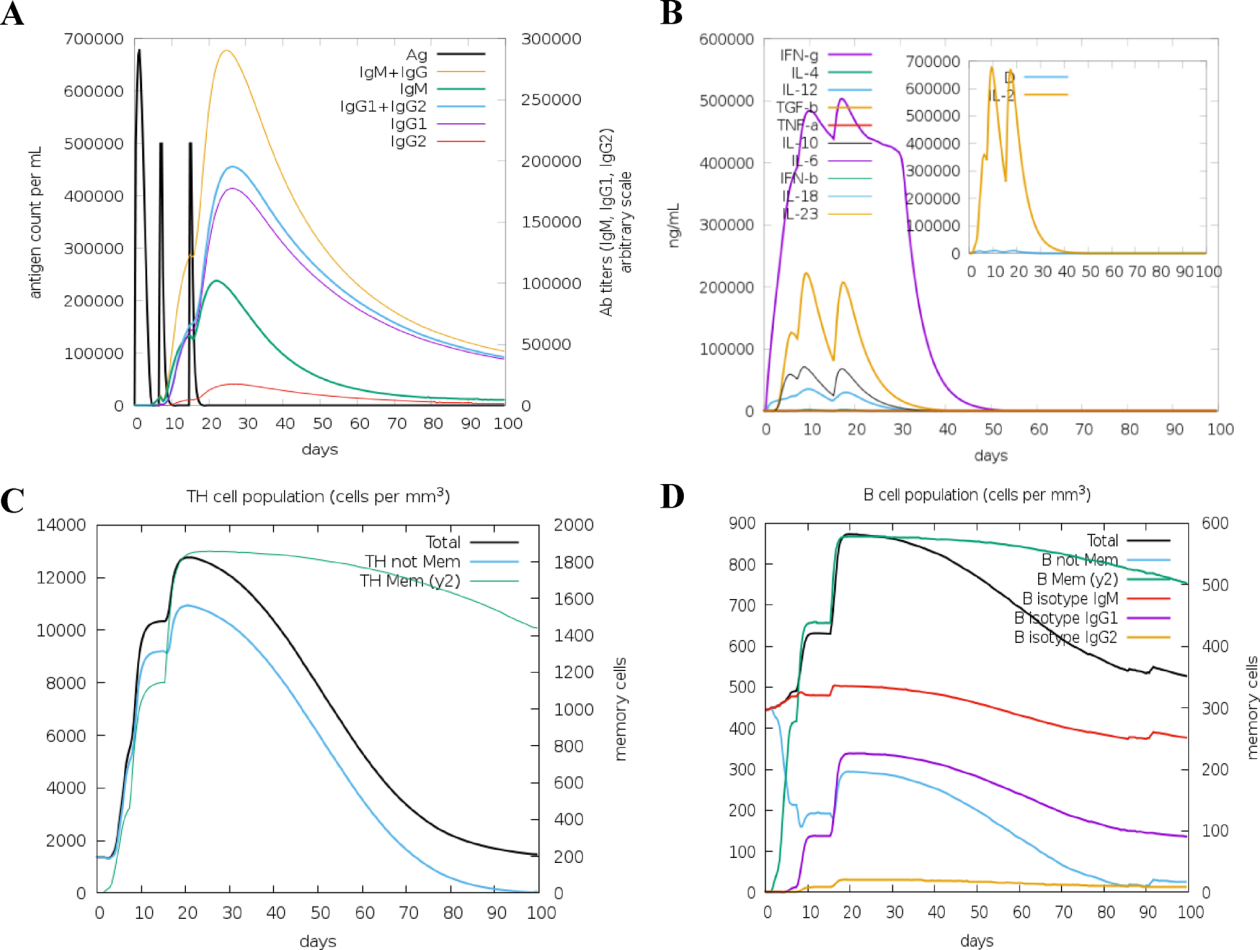
Immune response prediction generated by immunization with the TcGAPDH protein using the C-ImmSim server. (**A**) Titre and isotype of antibodies obtained; (**B**) Cytokine profile generated; (**C**, **D**) Increase in T and B-cell population and generation of memory cells

The immune response was evaluated by simulating immunization with three doses of the TcGAPDH protein, administered at seven-day intervals, and compared to a negative control with a short non-immunogenic peptide. The control simulation showed no predictive antibody production, with basal antibody levels, low cytokine production, and absence of memory cell differentiation (Figure S2), validating the platform’s baseline parameters. In contrast, TcGAPDH immunization elicited a robust immune response, evidenced by increased IgM and IgG titters with predominant IgG1 isotype expression and minimal expression of IgG2, indicative of a non-polarized Th1-Th2 response (Fig. 3). However, the simulation predicted elevated production of Th1 cytokines, including IFN-γ, IL-2, and IL-12. No production of Th2 cytokine as IL-4 was observed. Additionally, expansion of both B and T lymphocyte populations was observed, including the generation of immunological memory cell subsets.

### Refined Protein Structures for Molecular Docking and Molecular Dynamics Simulations

**Fig. 4:**
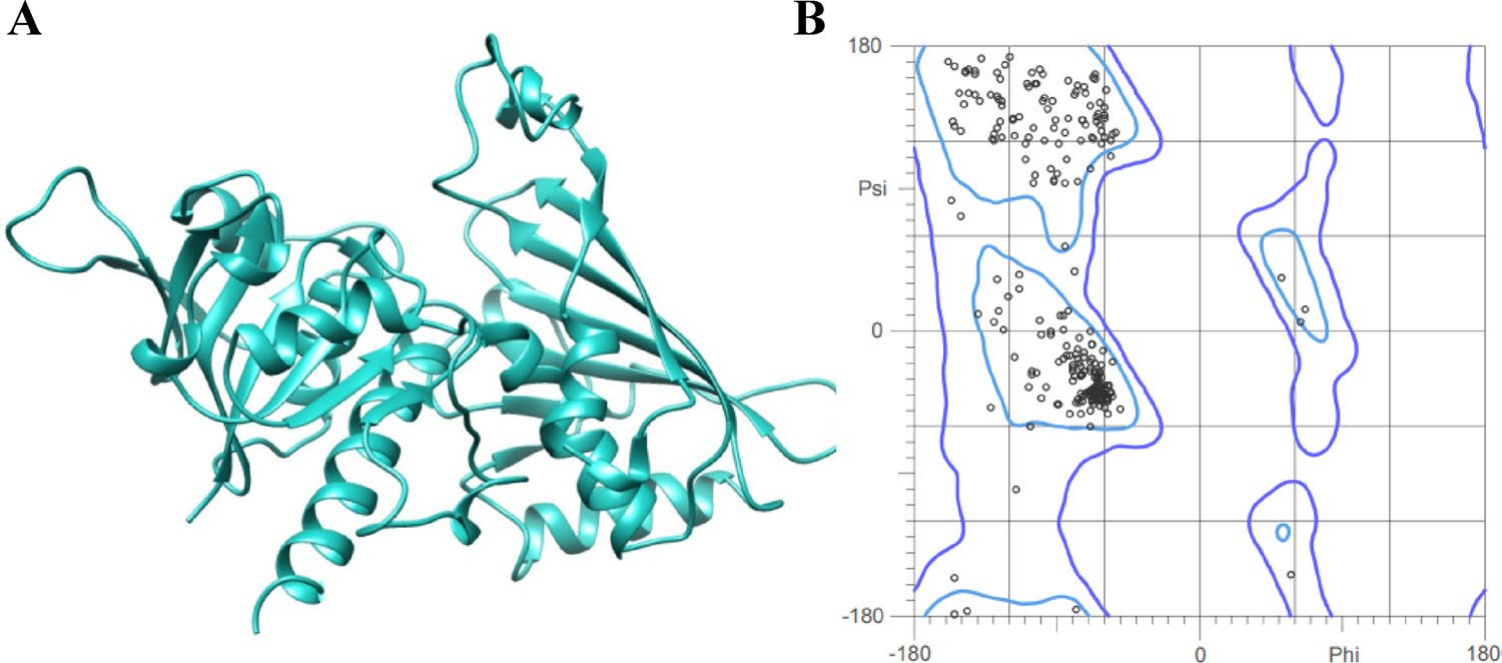
Three-dimensional structural assessment of the TcGAPDH protein. (**A**) Refined three-dimensional structure. (**B**) Ramachandran plot analysis of the refined TcGAPDH protein

The crystallographic structures of the TcGAPDH protein, TLR-2 and TLR-4 receptors, and control proteins SSL3 and MD-2 were systematically prepared and refined using UCSF Chimera software before molecular docking and molecular dynamics simulations. Structural assessment performed with MolProbity demonstrated that all processed structures exhibited reduced scores, indicating enhanced model quality compared to the original structures. The refined three-dimensional structure of the TcGAPDH protein, which is the protein of interest in this study, is presented in Fig. 4 along with its corresponding Ramachandran plot to demonstrate structural validation. As observed, 95.2% and 99.7% of amino acid residues were positioned within the favoured and allowed regions, respectively, demonstrating acceptable stereochemical parameters. The relevant evaluation parameters of all refined models are presented in the supplementary material.

### Molecular Docking Simulations

To elucidate the three-dimensional structural conformations, identify putative binding sites, and evaluate the binding affinities important for the interactions between TcGAPDH and the membrane-associated Toll-like receptors TLR-2 and TLR-4, molecular docking simulations were performed using the HDOCK server. This analysis demonstrated that TcGAPDH exhibited adequate molecular docking with TLR-2 and TLR-4 receptors, indicating a favourable interaction based on platform scoring.

### TcGAPDH-TLR2 Molecular Docking Analysis

For the molecular docking analysis between the TcGAPDH and TLR-2 receptor, the Staphylococcal Superantigen-Like protein 3 (SSL3) from *Staphylococcus aureus*, which functions as a TLR-2 antagonist, was employed as a reference standard in parallel molecular docking analyses with TLR-2 under identical computational parameters and conditions. The SSL3-TLR2 reference docking generated ten predicted complexes with an average docking score of -242.9 ± 34.6, which were analysed using PDBsum to obtain a detailed visualization of the intermolecular interactions. Subsequently, the TcGAPDH-TLR2 molecular docking was carried out, yielding ten predicted complexes with an average docking score of -263.8 ± 7.1. These complexes were equally analysed using PDBsum to validate the results against the reference standard.

**Fig. 5:**
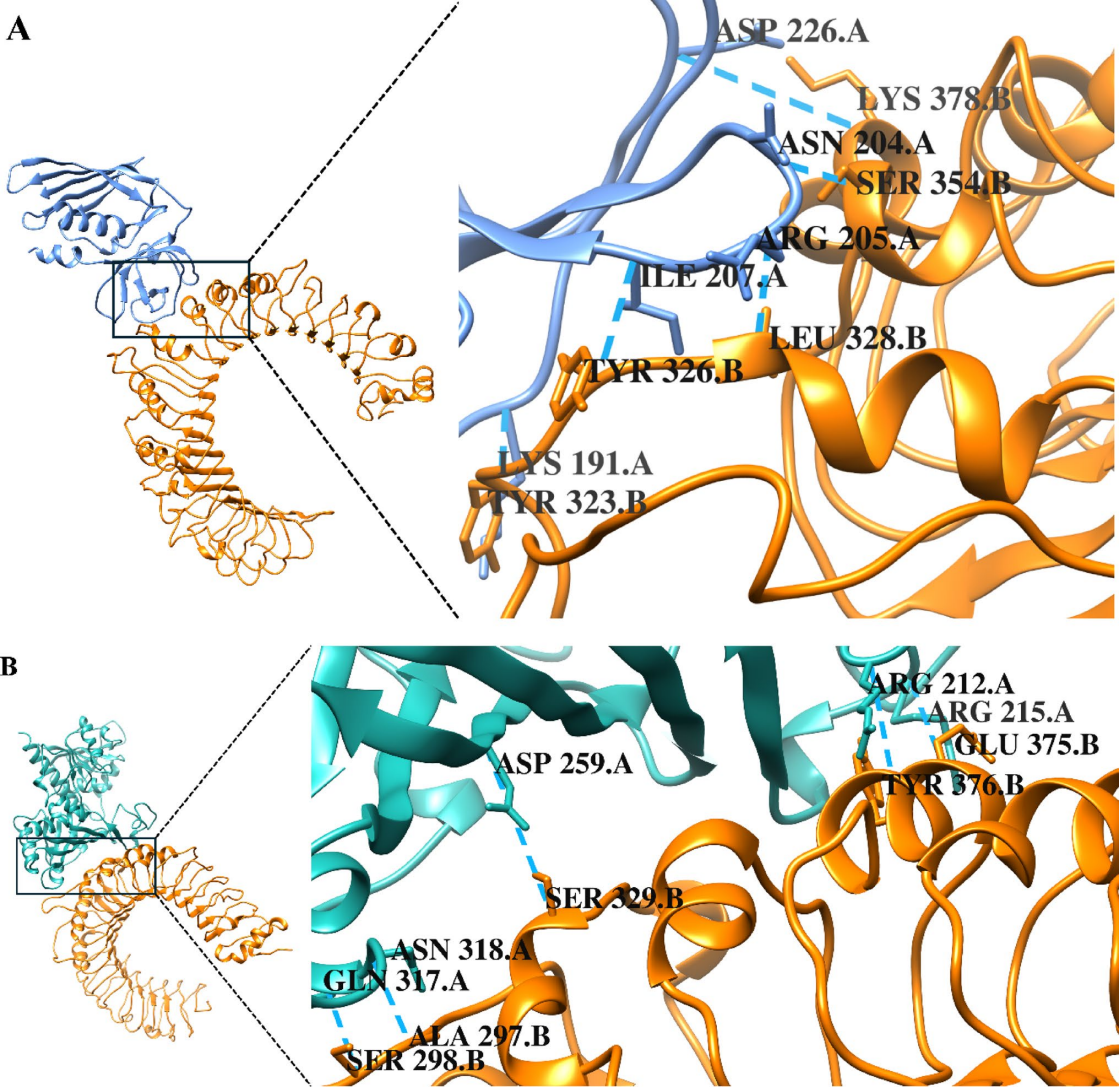
Three-dimensional structures of TLR-2 (orange) complexes generated through molecular docking analysis using the HDOCK server. (**A**) SSL3 (blue)-TLR2 reference complex (PDB ID: 5D3I). (**B**) TcGAPDH (green; PDB ID:3IDS)-TLR2(PDB ID:2Z7X) complex. Zoomed-in views of the binding interface between SSL3 and TcGAPDH with TLR2 are displayed, with residues participating in hydrogen bonding interactions marked.Structures were visualized using USCF Chimera 1.18

**Fig. 6:**
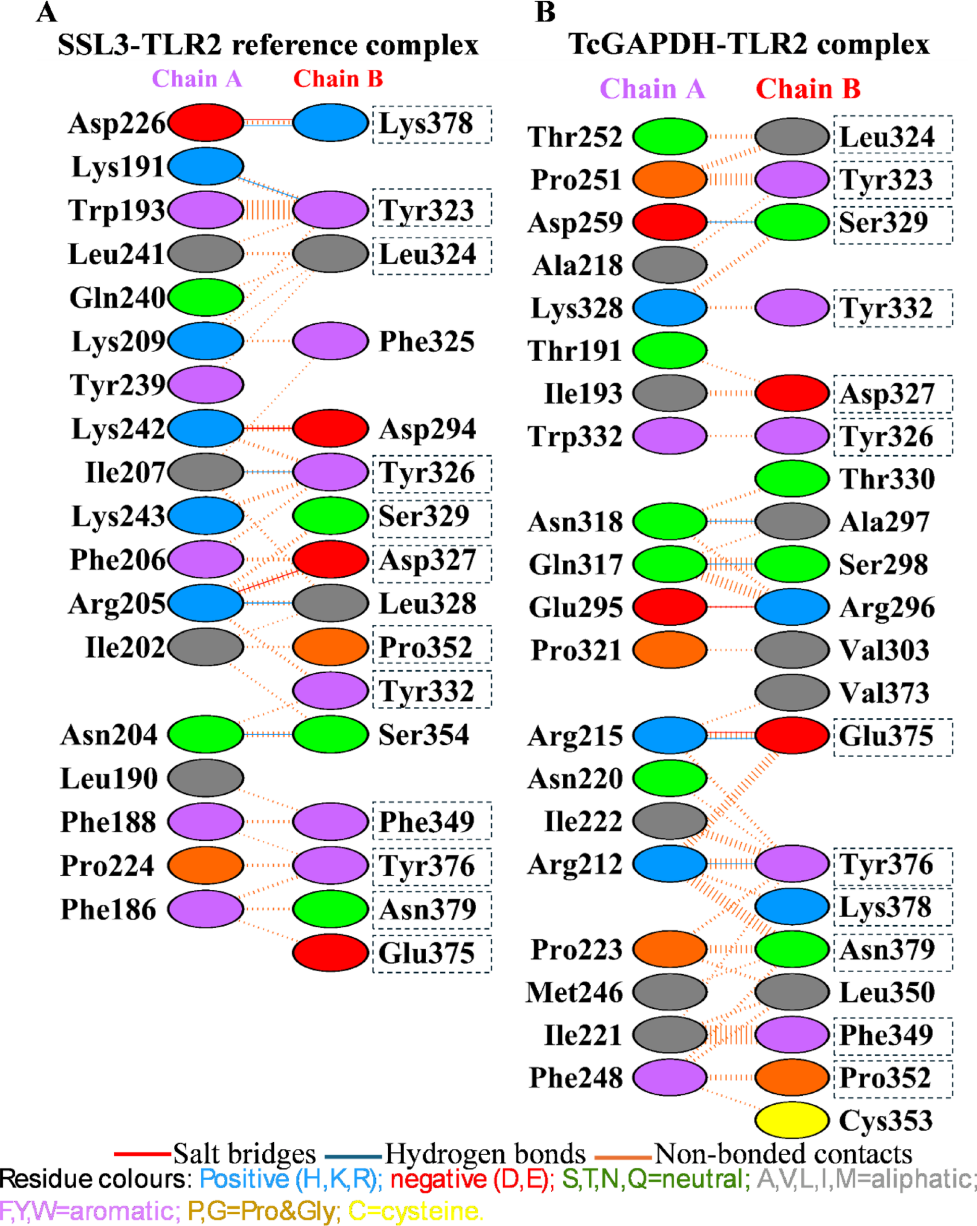
Intermolecular interaction analysis of TLR-2 complexes using PDBsum. (**A**) SSL3(chain A) – TLR2(chain B) reference complex showing amino acid contacts, hydrogen bonds, salt bridges and non-bonded contacts. (**B**) TcGAPDH (chain A)–TLR2(chain B) complex showing amino acid contacts, hydrogen bonds, salt bridges and non-bonded contacts. Conserved TLR-2 binding residues are inside dashed boxes. Figures were edited to show the correct amino acid residue numbering according to the canonical sequence

For subsequent analysis, two complexes were selected: the SSL3-TLR2 reference complex with the lowest docking score of -338.0, illustrated in Fig. 5A, and the TcGAPDH-TLR2 complex exhibiting the lowest docking score of -276.5, illustrated in Fig. 5B. The TcGAPDH-TLR2 complex was selected based on its structural conformational similarity to the reference complex, the presence of favourable hydrogen bond interactions and the conservation of amino acid residues between the reference and the complex within the binding site (Fig. 6).

PDBsum analysis of the two complexes selected revealed detailed amino acid contact maps, identifying the specific residues involved in the TcGAPDH -TLR2 interaction. In the reference complex, 18 amino acid residues from SSL3 formed direct contacts with 16 TLR-2 residues, stabilized by five hydrogen bonds, three salt bridges, and 155 non-bonded contacts. For the TcGAPDH-TLR2 complex, 20 amino acid residues from TcGAPDH were in direct contact with 20 TLR-2 residues, with five hydrogen bonds, two salt bridges, and 139 non-bonded contacts stabilizing the interface.

Notably, comparative analysis revealed that several TLR-2 residues involved in TcGAPDH binding were conserved in the reference complex interaction. These shared residues include Tyr323, Leu324, Tyr326, Asp327, Ser329, Tyr332, Phe349, Pro352, Glu375, Tyr376, Lys378, and Asn379, which participate in interactions of both complexes (Fig. 6). The corresponding docking scores and the number of intermolecular interactions for both selected complexes are summarized in Table 7.

**Table 7:** Docking scores and interface statistics were obtained for each complex selected using the HDOCK and PDBsum servers

Parameter	SSL3-TLR2 reference complex	TcGAPDH-TLR2 complex	MD2-TLR4 reference complex	TcGAPDH-TLR4 complex
Docking score	− 338.0	− 276.5	− 400.2	− 370.0
No. of interface residues	18/16	20/20	20/29	39/49
No. of salt bridges	3	2	5	3
No. of hydrogen bonds	5	5	25	9
No. of non-bonded contacts	155	139	190	331

### TcGAPDH-TLR4 Molecular Docking Analysis

The molecular docking analysis between TcGAPDH and TLR-4 receptor was conducted following the same computational approach employed for TLR-2, where the lymphocyte antigen 96 (MD-2) protein, which serves as a co-receptor for TLR-4 signalling, was utilized as a reference standard under identical docking parameters. The MD2-TLR4 reference docking generated ten predicted complexes with an average docking score of -331.8 ± 26.2, which were analysed using PDBsum to establish baseline intermolecular interactions. Afterwards, the TcGAPDH-TLR4 molecular docking was performed, yielding ten predicted complexes with an average docking score of -308.2 ± 26.0. These complexes were equally analysed using PDBsum to validate the results against the reference standard.

**Fig. 7:**
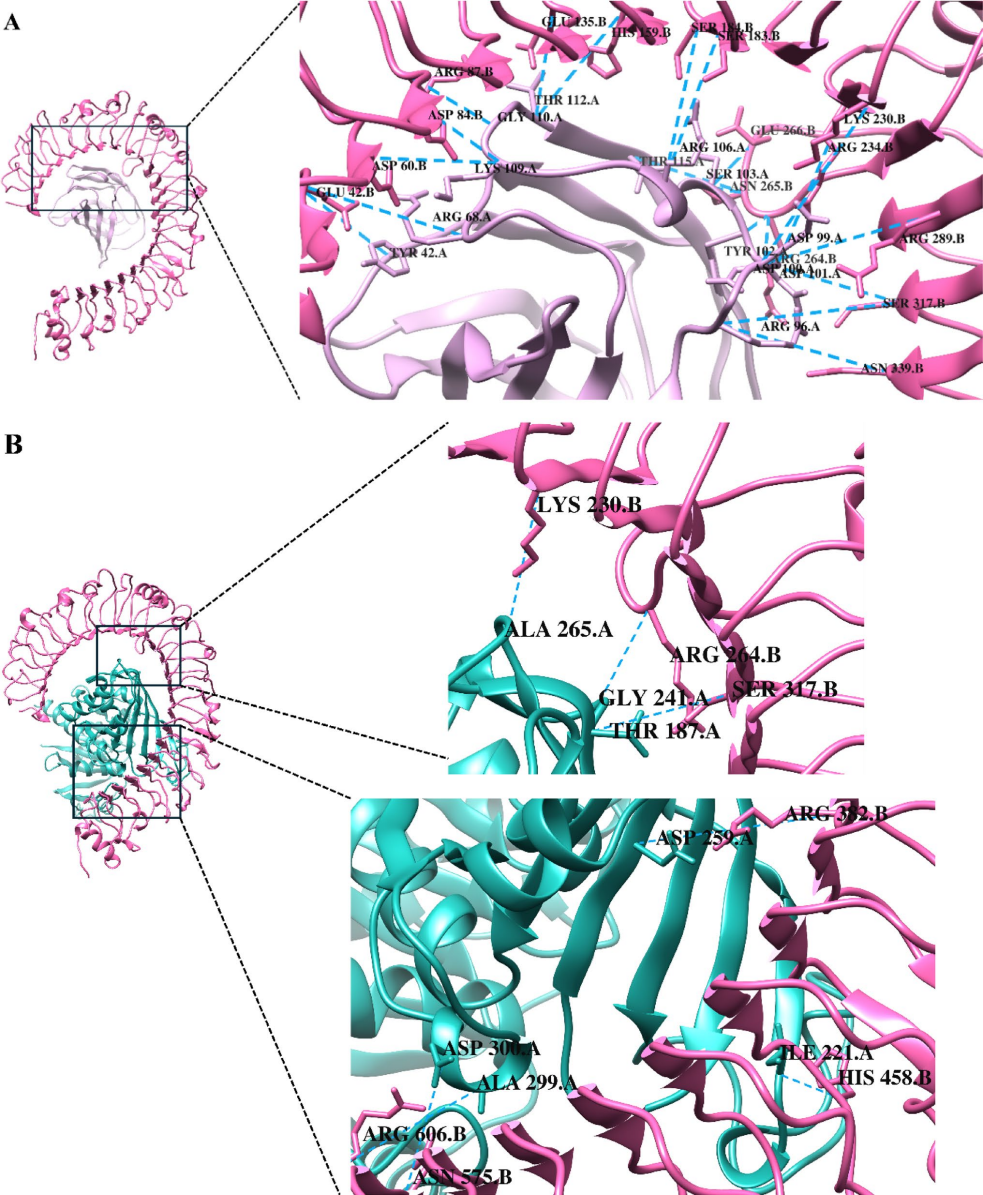
Three-dimensional structures of TLR-4 (pink) complexes generated through molecular docking analysis using the HDOCK server. (**A**) MD-2 (purple)-TLR4 reference complex (PDB ID: 4G8A). (**B**) TcGAPDH (green; PDB ID: 3IDS)-TLR4 (PDB ID: 4G8A) complex. Zoomed-in views of the binding interface between MD-2 and TcGAPDH with TLR4 are displayed, with residues participating in hydrogen bonding interactions marked Structures were visualized using USCF Chimera 1.18

**Fig. 8:**
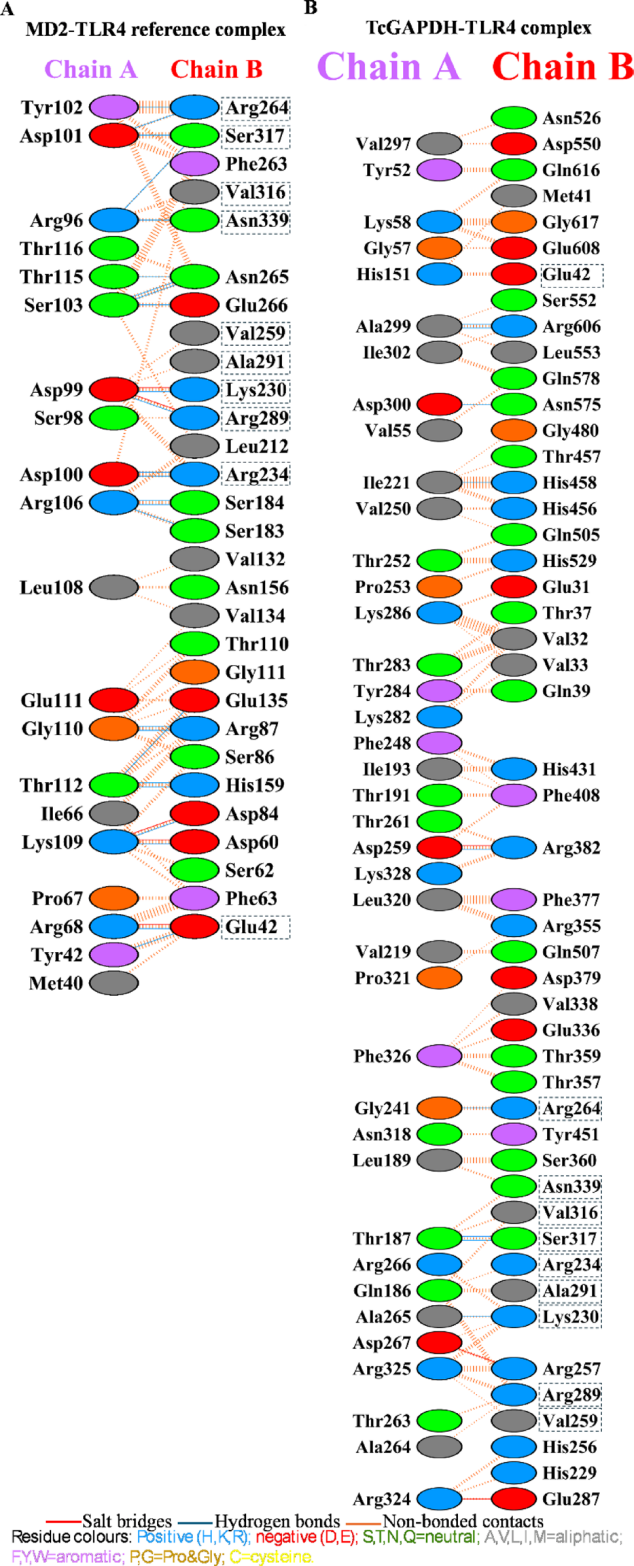
Intermolecular interaction analysis of TLR-4 complexes using PDBsum. (**A**) MD-2(chain A) – TLR4(chain B) reference complex showing amino acid contacts, hydrogen bonds, salt bridges and non-bonded contacts. (**B**) TcGAPDH (chain A) – TLR4(chain B) complex showing amino acid contacts, hydrogen bonds, salt bridges and non-bonded contacts. Conserved TLR-4 binding residues are inside dashed boxes. Figures were edited to show the correct amino acid residue numbering according to the canonical sequence

Based on the docking results, two complexes were selected for subsequent analysis: the MD2-TLR4 reference complex with the lowest docking score of -400.2, illustrated in Fig. 7A, and the TcGAPDH-TLR4 complex showing the lowest docking score of − 370.0, illustrated in Fig. 7B. The TcGAPDH-TLR4 complex was also selected based on its structural conformational similarity to the reference complex, the presence of favourable hydrogen bond interactions and the conservation of amino acid residues between the reference and the complex within the binding site (Fig. 8).

PDBsum analysis of the selected complexes revealed detailed amino acid contact maps for both TcGAPDH -TLR4 interactions. In the MD2-TLR4 reference complex, 20 amino acid residues from MD-2 formed direct contacts with 29 TLR-4 residues, with 25 hydrogen bonds, five salt bridges, and 190 non-bonded contacts. For the TcGAPDH-TLR4 complex, 39 amino acid residues from TcGAPDH were in direct contact with 49 TLR-4 residues, stabilized by nine hydrogen bonds, three salt bridges, and 331 non-bonded contacts.

Interestingly, a comparative analysis showed that several TLR-4 residues involved in TcGAPDH binding were conserved in the reference complex interaction. These shared residues include Glu42, Lys230, Arg234, Val259, Arg264, Arg289, Ala291, Val316, Ser317, and Asn339 (Fig. 8). The docking scores and number of intermolecular interactions for both selected complexes are summarized in detail in Table 7.

### Molecular Dynamics Simulations

To explore the structural and dynamic behaviour of the selected protein complex, 300 ns molecular dynamics simulations were performed on both TcGAPDH-TLR2 and TcGAPDH-TLR4 systems. The analysis demonstrated that, following the initial docking conformation, structural variation of both complexes remained constant, with consistent intermolecular interactions and binding affinities maintained throughout the simulation period.

### TcGAPDH-TLR2 Molecular Dynamic Simulation

**Fig. 9:**
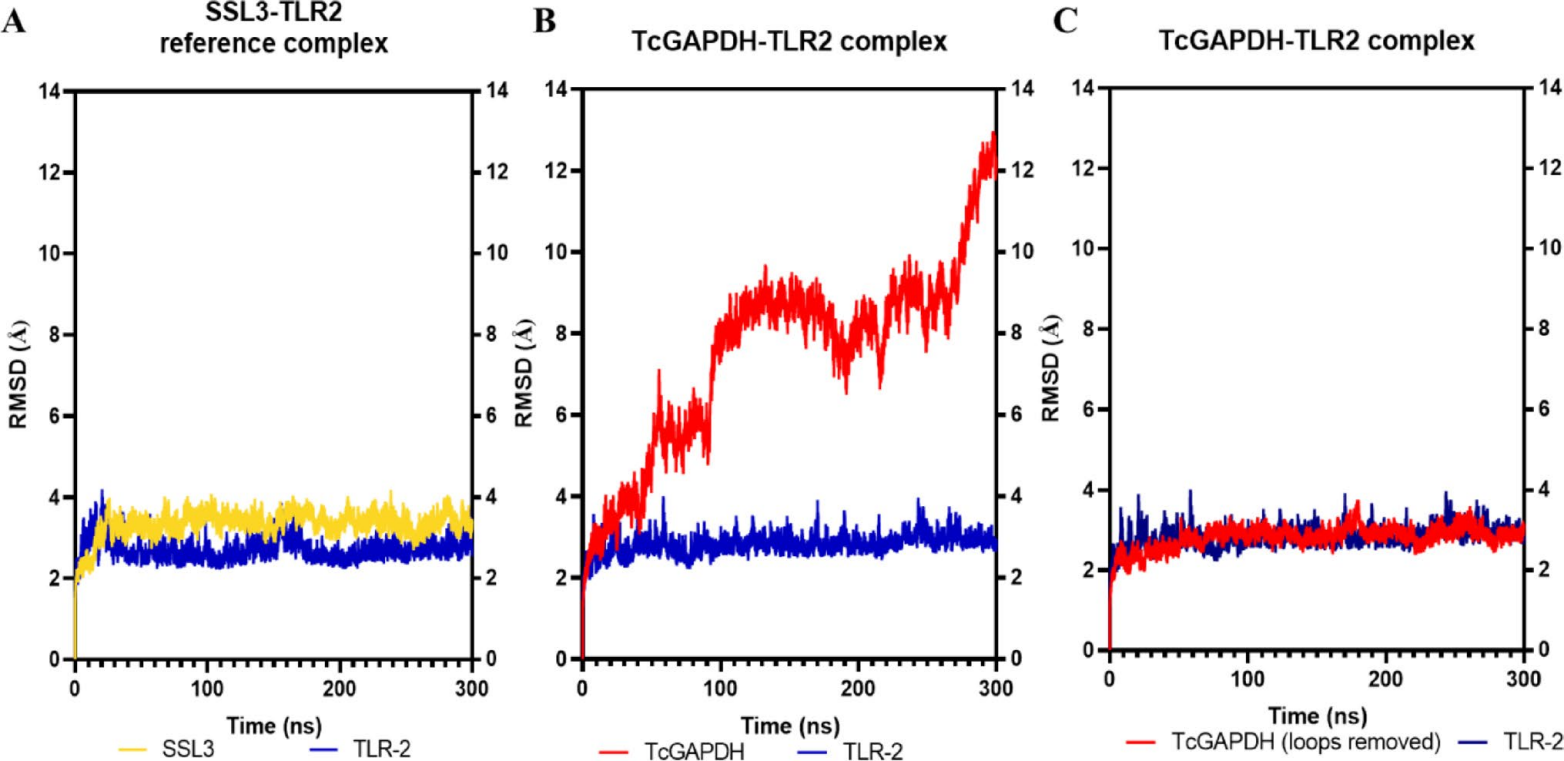
Structural variation analysis of TLR-2 receptor (blue) and ligands TcGAPDH (red) and SSL3 (yellow). (**A**, **B**) The TLR-2 receptor exhibits consistent structural variation in both molecular complexes, a behaviour that is replicated in the SSL3 protein of the reference complex. In contrast, in B), the TcGAPDH protein exhibits higher conformational variability throughout the simulation, primarily due to the presence of mobile loops. Notably, in C), the RMSD calculated excluding loop regions of TcGAPDH reveals a consistent structural variation. Root mean square deviation (RMSD) was calculated with respect to the initial conformation (t = 0 ns) during 300 ns molecular dynamics trajectories using the cpptraj module of the AMBER Tools package

RMSD analysis results for the TcGAPDH-TLR2 molecular dynamics simulation are presented in Fig. 9. Comparative assessment with the SSL3-TLR2 reference complex (Fig. 9A) revealed that both the SSL3 and TLR-2 receptor maintained constant structural variation throughout the 300 ns simulation period, exhibiting average RMSD values of 3.3 ± 0.3 Å and 2.7 ± 0.3 Å, respectively.

In contrast, the RMSD profile of the TcGAPDH-TLR2 complex revealed that the TcGAPDH protein exhibited pronounced fluctuations, reflecting structural variability relative to the initial conformation, with an average of 7.5 ± 2.4 Å (Fig. 9B). This structural fluctuation is characterized by minimal deviations during the initial 100 ns, with reduced variation between 100–200 ns, and peak values during the final 100 ns of the simulation period. Visual inspections indicated that this behaviour was mainly attributed to highly mobile loop regions and intrinsically disordered segments within the protein folding. To assess the structural integrity of the domain core, these loop regions were excluded, and a new RMSD calculation was performed. The new analysis demonstrated notable reduced structural variation, with RMSD values exhibiting minimal fluctuation throughout the simulation period, averaging 2.8 ± 0.3 Å (Fig. 8C). Conversely, the TLR-2 receptor maintains structural variation (with an average RMSD of 2.8 ± 0.2 Å), related to its conformational behaviour observed in the reference complex.

**Fig. 10:**
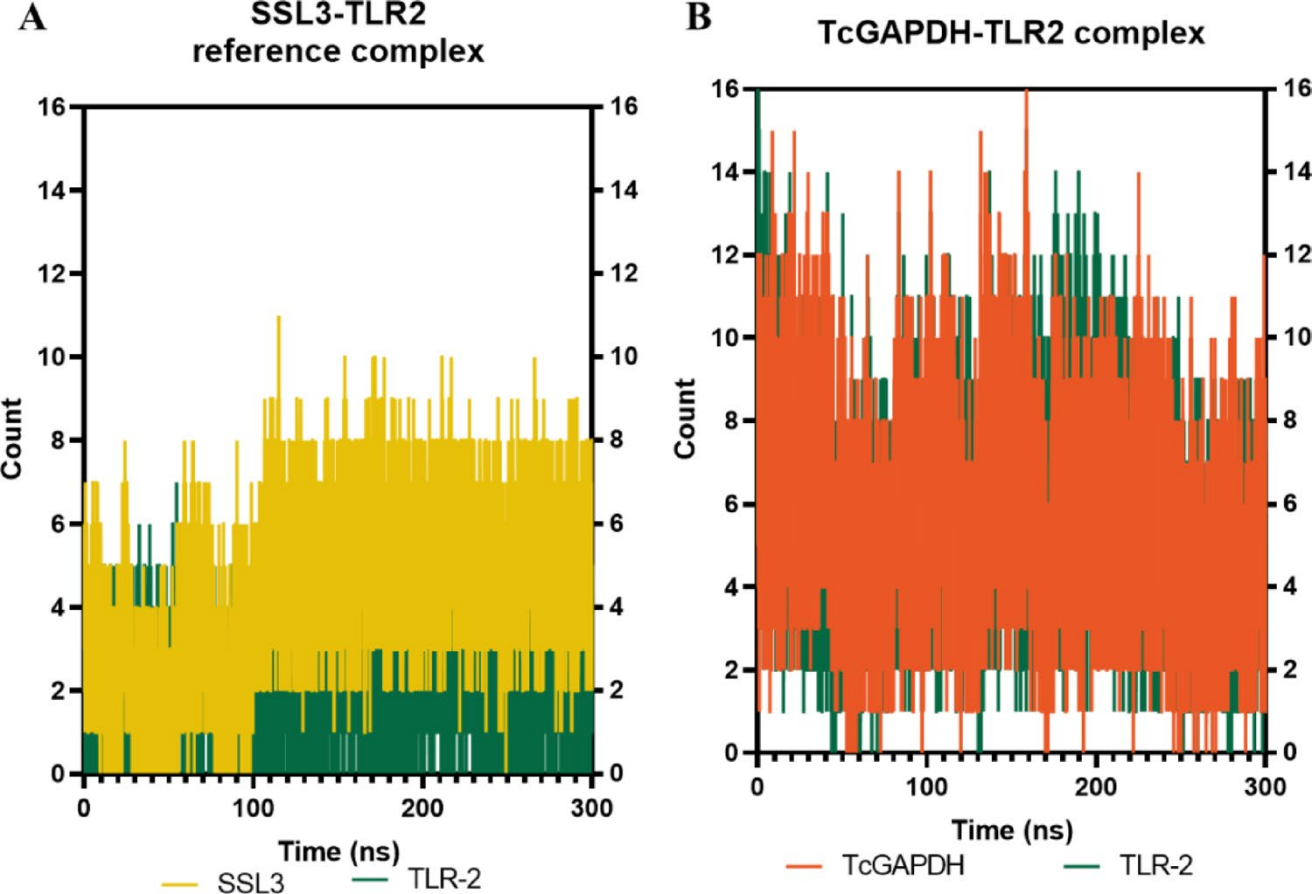
Quantitative analysis of hydrogen bond interactions between TLR-2 receptor (green) and TcGAPDH protein (orange). The number of hydrogen bonds remains relatively constant throughout the simulation, exhibiting similar behaviour to the reference complex that presents SSL3 protein (yellow) as ligand. The analysis was performed using 300 ns molecular dynamics trajectories generated with the cpptraj module of the AMBER Tools package. The graphs represent: (**A**) the SSL3-TLR2 reference complex, and (**B**) the TcGAPDH-TLR2 complex

Analysis of hydrogen bond interactions (Fig. 10) reveals that in the reference complex (Fig. 10A), both the SSL3 and TLR-2 maintain hydrogen bond formation throughout the simulation period, with an average of 4 and 2 hydrogen bonds, respectively, and a modest increase observed after the initial 100 ns. The TcGAPDH-TLR2 complex (Fig. 10B) exhibits a higher frequency of hydrogen bond formation (averaging 5 and 6 interactions, respectively); these interactions remain stable throughout the 300 ns simulation, displaying behaviour analogous to the reference complex.

**Fig. 11:**
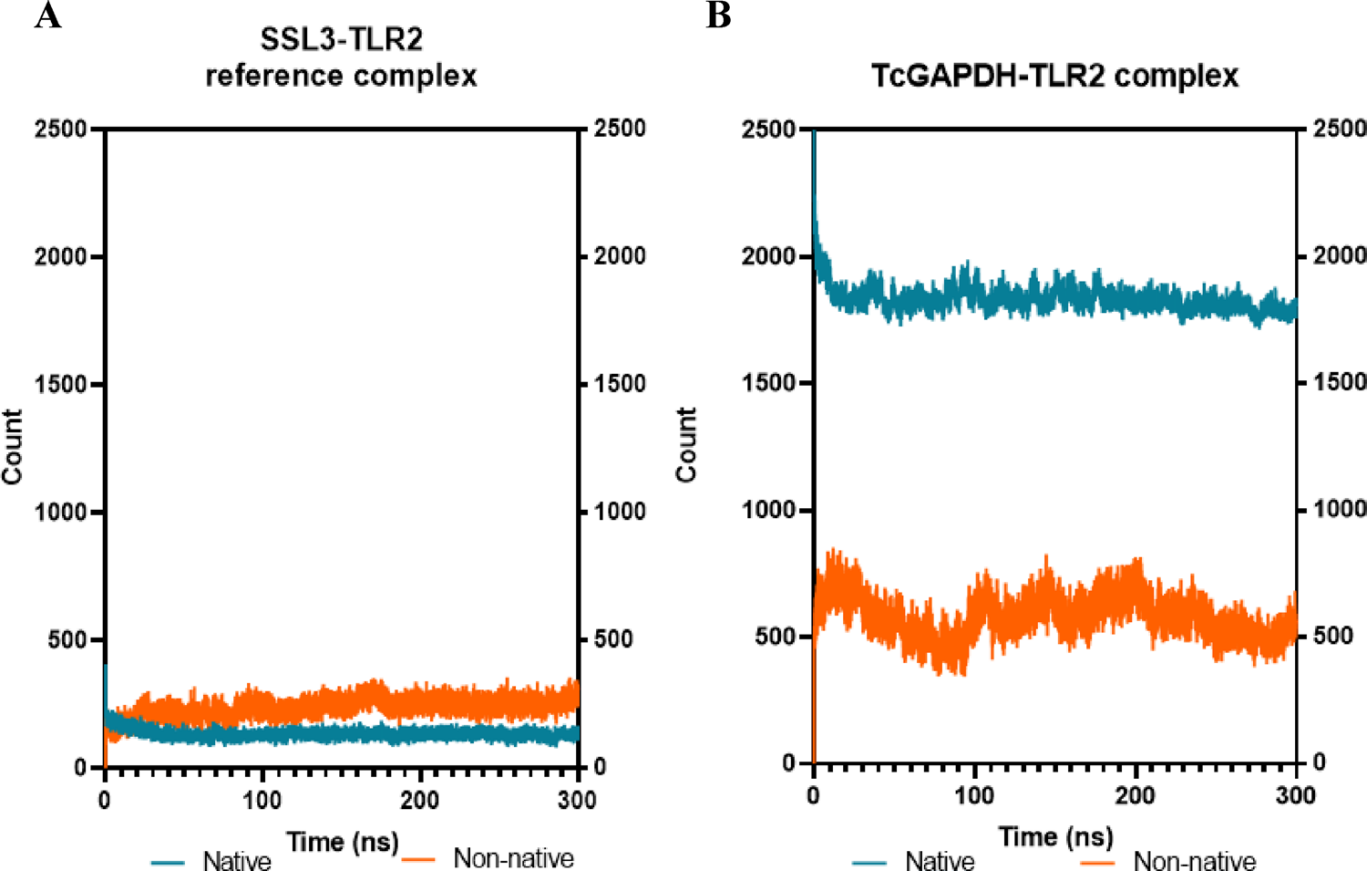
Quantitative analysis of native (green) and non-native (orange) contacts between TLR-2 receptor and TcGAPDH protein. The number of intermolecular contacts remains relatively constant throughout the simulation, exhibiting similar behaviour to the reference control complex. The analysis was performed using 300 ns molecular dynamics trajectories generated with the cpptraj module of the AMBER Tools package. The graphs represent: (**A**) the SSL3-TLR2 reference complex, and (**B**) the TcGAPDH-TLR2 complex

Native and non-native contacts analysis (Fig. 11) indicates that the reference complex (Fig. 11A) maintains contacts count throughout the simulation, with average values of 135 ± 18 and 238 ± 36 for native and non-native contacts, respectively. Although the TcGAPDH–TLR-2 complex (Fig. 11B) demonstrates a higher number of both native and non-native contacts (averaging 1835 ± 48 and 580 ± 79, respectively), these remain throughout the simulation, in line with the behaviour observed in the reference complex.

**Fig. 12:**
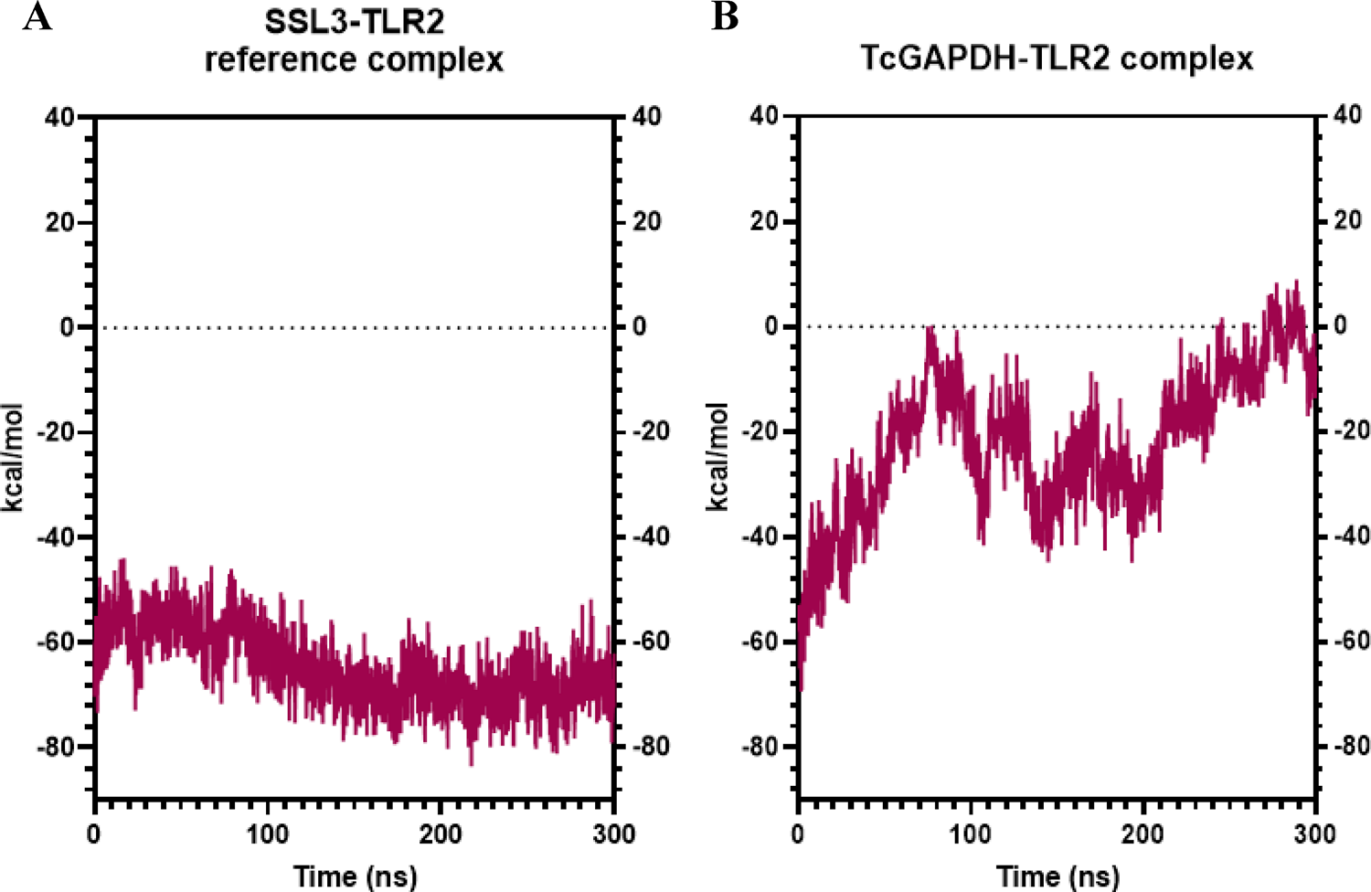
Binding affinity analysis between TLR-2 receptor and TcGAPDH protein using the MM/GBSA (Molecular Mechanics/Generalized Born Surface Area) method. The analysis reveals slightly variable behaviour of the TcGAPDH-TLR2 complex compared to the reference complex, which presents consistent and thermodynamically favourable binding affinity. The binding free energy was calculated throughout 300 ns molecular dynamics trajectories using the cpptraj module of the AMBER Tools package. The graphs represent: (**A**) the SSL3-TLR2 reference complex, and (**B**) the TcGAPDH-TLR2 complex

Binding free energy analysis reveals that the TcGAPDH-TLR2 complex (Fig. 12A) exhibits moderately fluctuating behaviour with consistently negative values (average of -22.01 ± 13.6 kcal/mol) throughout the simulation. This contrasts with the reference complex (Fig. 12B), which demonstrates less fluctuation behaviour with an average binding free energy of -64.62 ± 7.2 kcal/mol, indicating better binding affinity. Despite these slight behavioural differences relative to the reference complex, the results indicate favourable interaction and binding affinity between the TcGAPDH and TLR-2 receptor.

Furthermore, the average values and standard deviations for RMSD, hydrogen bonds, contacts, and binding free energies of each complex are summarized in Table 8. These comprehensive data provide a quantitative overview of the structural behaviour and interactions persistence in both complexes throughout the simulation period.

**Table 8:** Average structural and energetic parameters of molecular complexes during molecular dynamics simulations

Parameter		SSL3-TLR2 reference complex	TcGAPDH-TLR2 complexes	MD2-TLR4 refence complex	TcGAPDH-TLR4 complexes
RMSD (Å)	Receptor	2.7 ± 0.3	2.8 ± 0.2	3.0 ± 0.4	3.2 ± 0.4
	Ligand	3.3 ± 0.3	7.5 ± 2.4	3.6 ± 0.3	4.2 ± 0.9
Hydrogen bond	Receptor	2 ± 1	6 ± 2	8 ± 2	7 ± 2
	Ligand	4 ± 2	5 ± 2	5 ± 2	8 ± 2
Contacts	Native	135 ± 18	1835 ± 48	185 ± 19	125 ± 43
	Non-native	238 ± 36	580 ± 79	236 ± 30	572 ± 85
MMGBSA (kcal/mol)		− 64.62 ± 7.2	− 22.01 ± 13.6	− 52.84 ± 6.8	− 49.76 ± 11.5

### TcGAPDH-TLR4 Molecular Dynamics Simulation

**Fig. 13:**
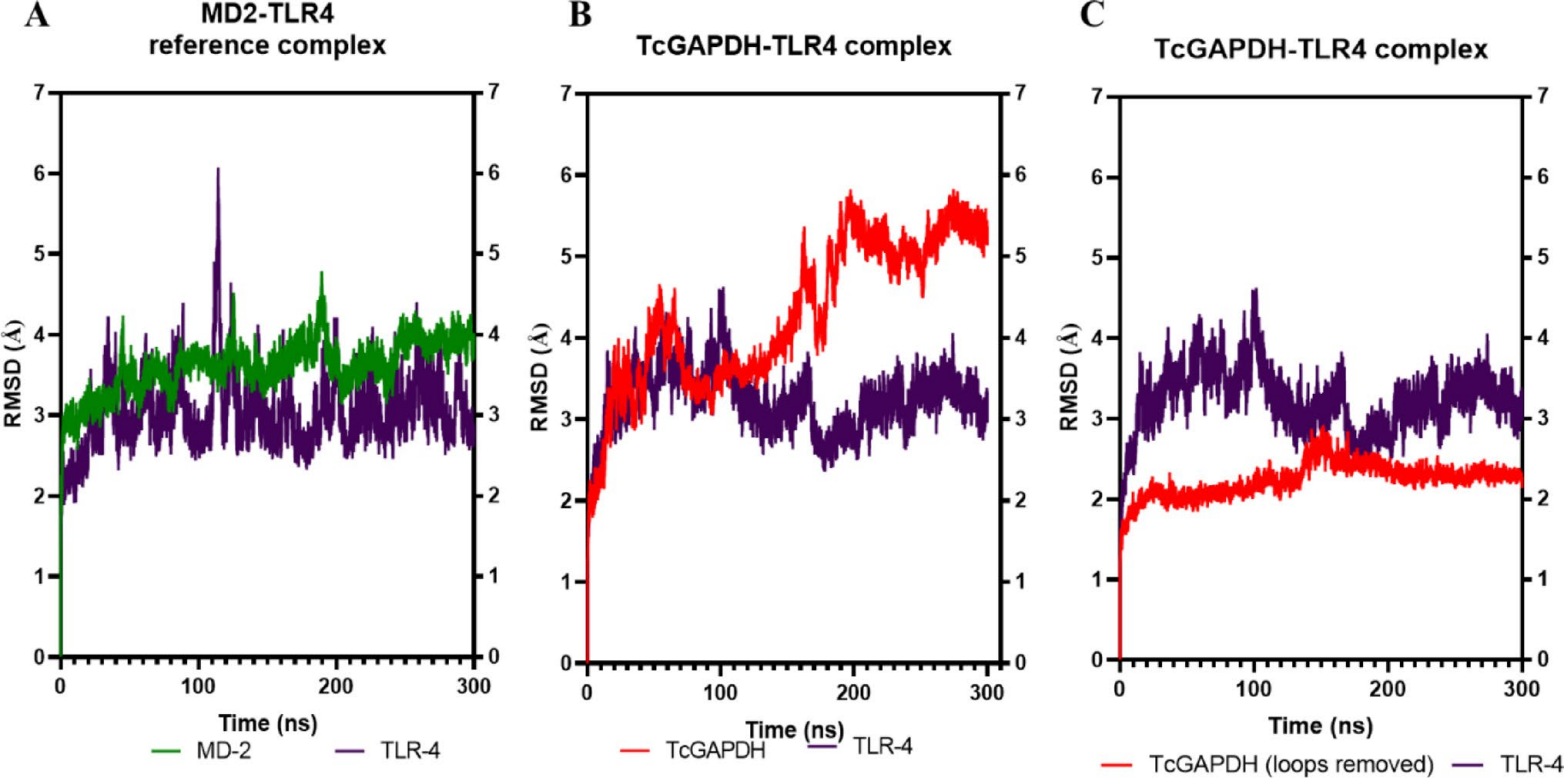
Structural variation analysis of TLR-4 receptor (purple) and ligands TcGAPDH (red) and MD-2 (green). (**A**, **B**) The TLR-4 receptor exhibits consistent structural variation in both molecular complexes, a behaviour that is replicated in the MD-2 protein of the reference complex. In contrast, in B), the TcGAPDH protein exhibits higher conformational variability throughout the simulation, primarily due to the presence of mobile loops. Notably, in (**C**), the RMSD calculated excluding loop regions of TcGAPDH reveals a consistent structural variation. Root mean square deviation (RMSD) was calculated with respect to the initial conformation (t = 0 ns) during 300 ns molecular dynamics trajectories using the cpptraj module of the AMBER Tools package

For the TcGAPDH-TLR4 molecular dynamics simulations, the RMSD analysis results are presented in Fig. 13. Comparative assessment with the MD2-TLR4 reference complex (Fig. 13A) reveals that both the MD2 protein and the TLR-4 receptor exhibit consistent structural variation throughout the 300 ns simulation period, with average RMSD values of 3.6 ± 0.3 Å and 3.0 ± 0.4 Å, respectively.

By contrast, the RMSD profile of the TcGAPDH-TLR4 complex revealed that TcGAPDH exhibited pronounced fluctuations, reflecting structural variability relative to the initial conformation throughout the 300 ns simulation, with an average of 4.2 ± 0.9 Å (Fig. 13B). As in TcGAPDH-TLR2 molecular dynamics, visual inspections indicated that this behaviour was mainly attributed to highly mobile loop regions and intrinsically disordered segments within the protein folding, and to assess the structural integrity of the domain core, these loop regions were excluded, and a new RMSD calculation was performed. The new analysis revealed a notably reduced structural variation, with RMSD values exhibiting minimal fluctuation throughout the simulation period, averaging 2.2 ± 0.2 Å (Fig. 13C). Conversely, the TLR4 receptor maintains structural variation (average RMSD of 3.2 ± 0.4 Å), related to its conformational behaviour observed in the reference complex.

**Fig. 14:**
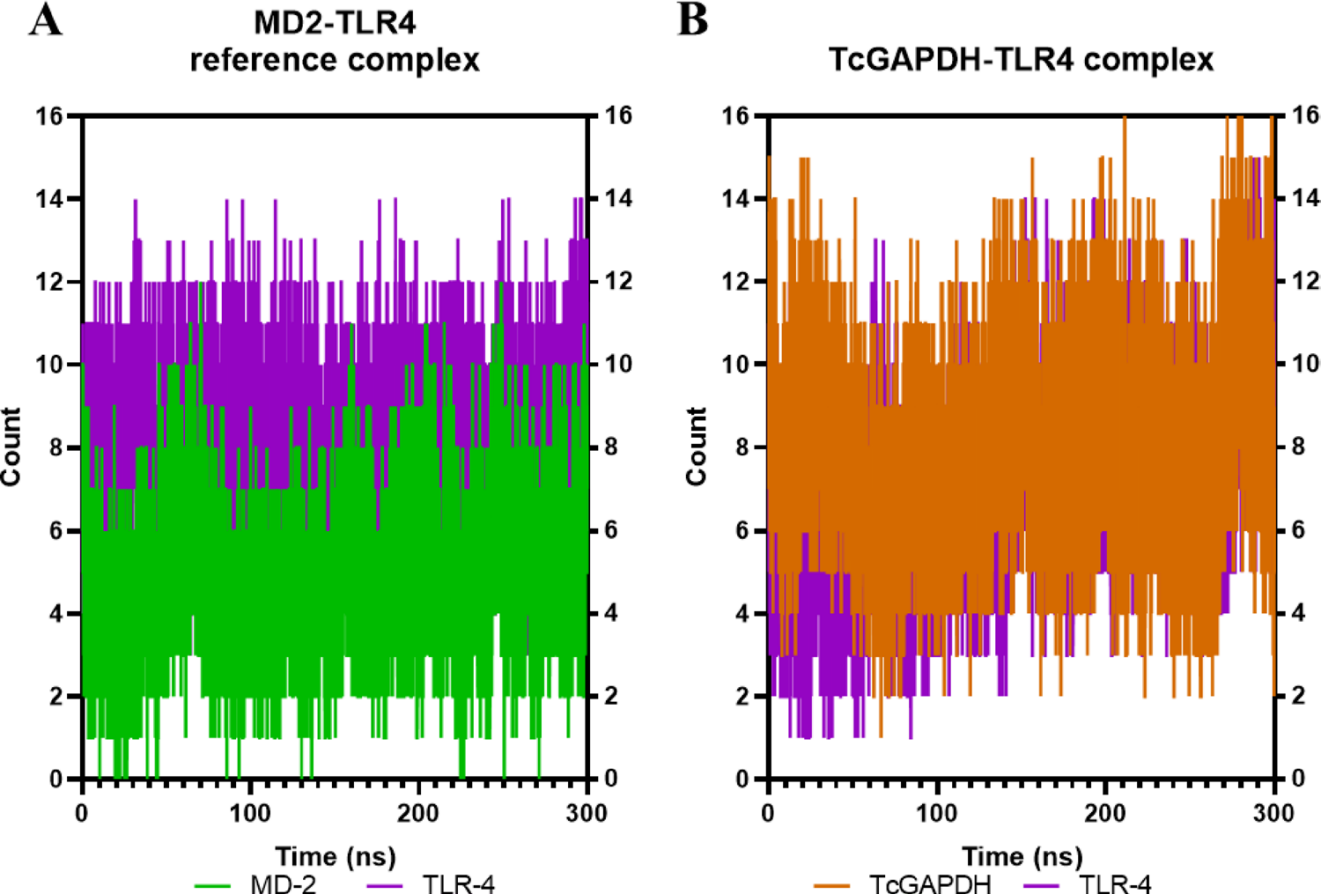
Quantitative analysis of hydrogen bond interactions between TLR-4 receptor (light purple) and TcGAPDH protein (orange). The number of hydrogen bonds remains relatively constant throughout the simulation, exhibiting similar behaviour to the control complex that presents the MD-2 protein (light green) as ligand. The analysis was performed using 300 ns molecular dynamics trajectories generated with the cpptraj module of the AMBER Tools package. The graphs represent: (**A**) MD2-TLR4 reference complex, (**B**) TcGAPDH-TLR4 complex

Analysis of hydrogen bond interactions (Fig. 14) shows that in the reference complex (Fig. 14A), both the MD-2 protein and TLR-4 maintain hydrogen bond formation throughout the simulation period, averaging 5 and 8 hydrogen bonds, respectively, with TLR-4 serving as the primary hydrogen bond donor. The TcGAPDH-TLR4 complex (Fig. 14B) demonstrates similarly stable hydrogen bond formation during the 300 ns simulation (averaging 8 and 7 hydrogen bonds, respectively), exhibiting behaviour analogous to the reference complex.

**Fig. 15:**
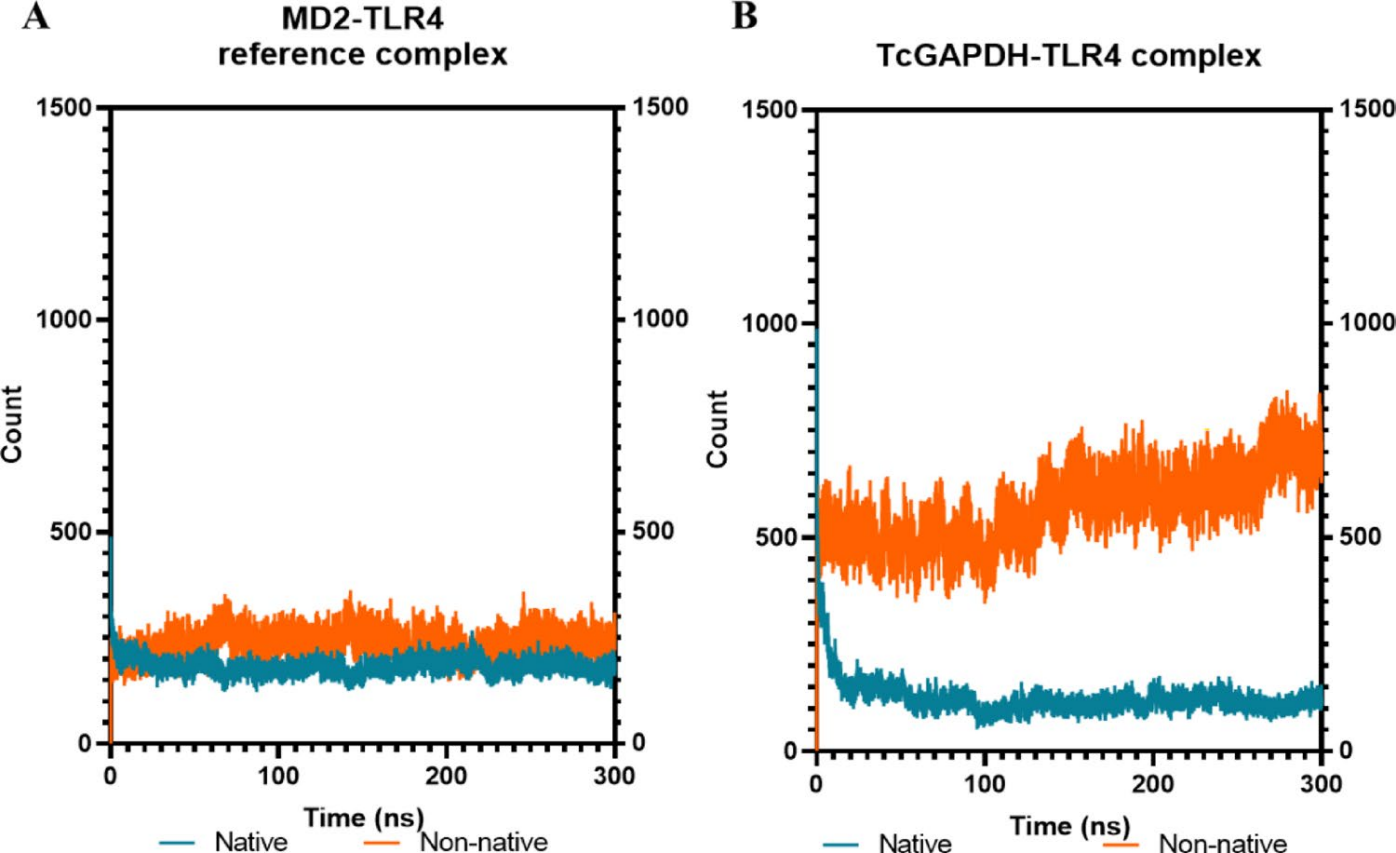
Quantitative analysis of native (green) and non-native (orange) contacts between the TLR-4 receptor and TcGAPDH protein. The number of intermolecular contacts remains relatively constant throughout the simulation, exhibiting similar behaviour to the reference control complex. The analysis was performed using 300 ns molecular dynamics trajectories generated with the cpptraj module of the AMBER Tools package. The graphs represent: (**A**) MD2-TLR4 reference complex, (**B**) TcGAPDH-TLR4 complex

Regarding native and non-native contact analysis (Fig. 15), the reference complex (Fig. 15A) maintains contacts throughout the simulation period, with average native and non-native contacts of 185 ± 19 and 236 ± 30, respectively. The TcGAPDH-TLR-4 complex (Fig. 15B) exhibits an elevated frequency of non-native contacts (with an average of 572 ± 85) compared to native contacts (with an average of 125 ± 43); however, both contact types remain consistent throughout the 300 ns simulation, in line with the reference complex behaviour.

**Fig. 16:**
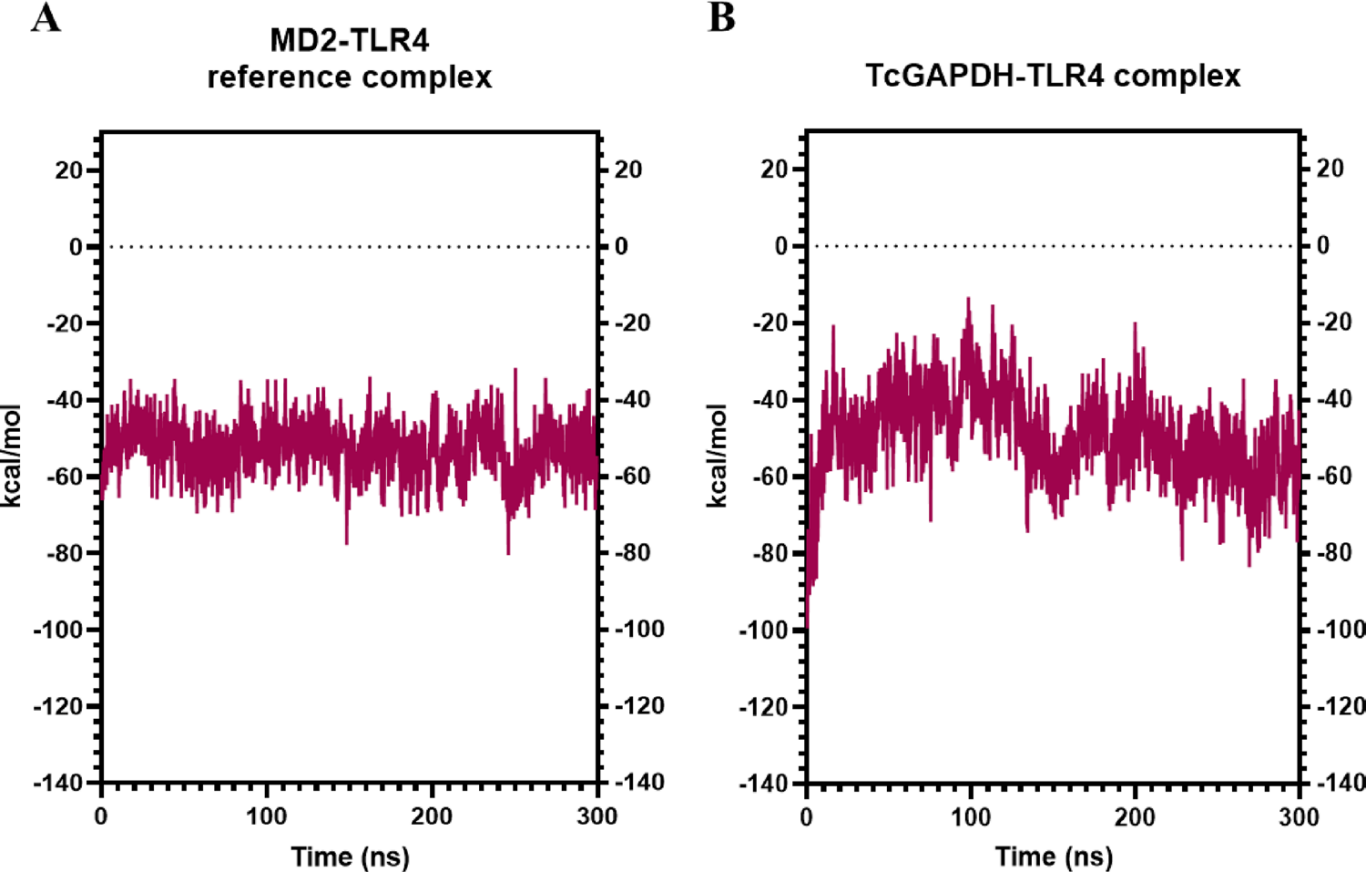
Binding affinity analysis between TLR4 receptor and TcGAPDH protein using the MM/GBSA (Molecular Mechanics/Generalized Born Surface Area) method. The analysis reveals similar behaviour of the TcGAPDH-TLR4 complex with respect to the reference complex, both exhibiting consistent and thermodynamically favourable binding affinity. The binding free energy was calculated throughout 300 ns molecular dynamics trajectories using the cpptraj module of the AMBER Tools package. The graphs represent: (**A**) MD2-TLR4 reference complex, (**B**) TcGAPDH-TLR4 complex

Binding free energy analysis reveals that the TcGAPDH-TLR4 complex (Fig. 16A) exhibits consistent behaviour with negative binding free energy values throughout the simulation (average of -49.76 ± 11.5 kcal/mol), comparable to the reference complex (Fig. 16B), which presented an average value of -52.84 ± 6.8 kcal/mol. These results indicate a favourable interaction and a strong binding affinity between TcGAPDH and TLR-4 receptor.

Furthermore, the average values and standard deviations for RMSD, hydrogen bonds, contacts, and binding free energies of each complex are summarized in Table 8. These comprehensive data provide a quantitative overview of the structural behaviour and interactions persistence in both complexes throughout the simulation period.

**Fig. 17:**
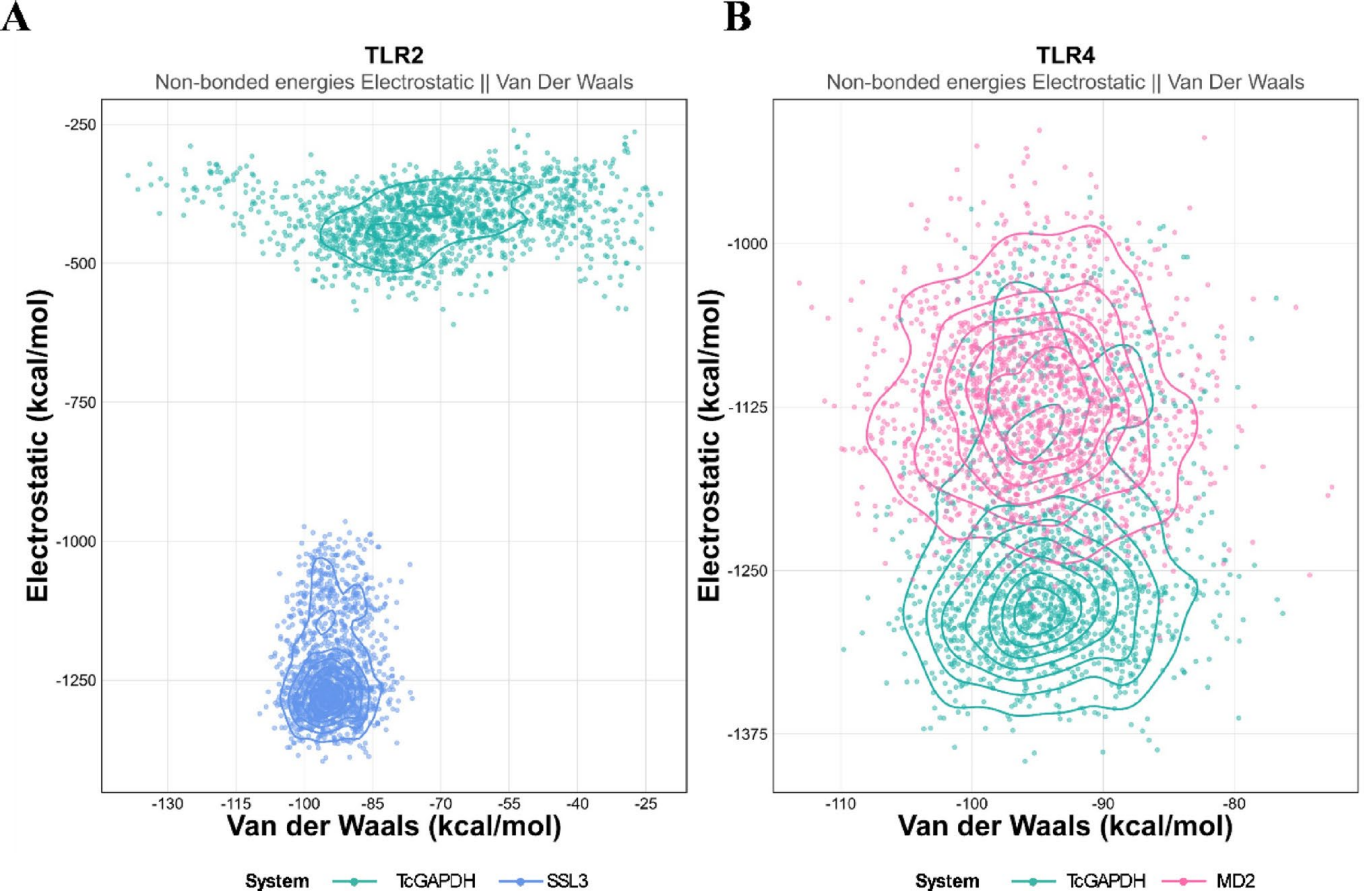
Non-bonded energy decomposition analysis (**A**) TLR2 complexes with SSL3 (reference, blue) and TcGAPDH (green). (**B**) TLR4 complexes with MD-2 (reference, pink) and TcGAPDH (green)

To dissect the energetic contributions to binding stability, we performed non-bonded energy decomposition analysis, separating electrostatic and Van der Waals components throughout the MD simulations (Fig. 17).

For the TLR2 system, the SSL3–TLR2 reference complex exhibited a high-affinity binding mode with electrostatic energies centered around -1000 to -1300 kcal/mol and Van der Waals contributions of -85 to -115 kcal/mol. The TcGAPDH–TLR2 complex displayed a distinct energetic profile with both electrostatic (-400 to -500 kcal/mol) and Van der Waals (-40 to -70 kcal/mol) interactions showing approximately twofold reduced magnitudes compared to SSL3.

For the TLR4 system, both the MD2–TLR4 reference complex and the TcGAPDH–TLR4 complex occupied overlapping regions in the energy landscape, with electrostatic energies ranging from -1000 to -1375 kcal/mol and Van der Waals contributions of -80 to -110 kcal/mol. In all systems, electrostatic interactions dominated the binding energetics, contributing approximately 90% of the total non-bonded energy.

## Discussion

Ten TcGAPDH coding sequences were identified, corresponding to strains belonging to DTUs I, II, and VI. The absence of sequences from the remaining DTUs (such as DTU III and IV) may be attributed to their predominantly sylvatic transmission cycles, which involve limited transmission to human populations, resulting in lower detection and sequencing of these genetic variants. In contrast, DTUs I, II, and VI have been widely analysed due to their direct association with domestic transmission cycles and their clinical relevance in human infections [9, 78, 79]. Comparative analysis of TcGAPDH amino acid sequences demonstrated a sequence identity percentage higher than 99.44% among all sequences analysed. This result is particularly significant, as it suggests that TcGAPDH maintains a highly conserved structure across different DTUs, which represents a desirable feature for vaccine candidate development.

Given the high degree of sequence conservation, a representative sequence was selected for further analysis. The chosen sequence corresponds to the Dm28c strain, classified within DTU I, which is the most widely distributed DTU across the Americas, maintaining both sylvatic and domestic transmission cycles [9]. Moreover, DTU I has been consistently associated with severe chronic manifestations of Chagas disease, particularly Chronic Chagasic Cardiomyopathy and severe meningoencephalitis [78]. These clinical associations reinforce the rationale for employing this sequence as a basis for exploring vaccine development, since it could potentially confer protection against the most severe clinical outcomes of the disease.

The analysis of the physicochemical, antigenic, and allergenic properties of TcGAPDH revealed favourable features for its evaluation as a potential vaccine candidate. This protein exhibited a theoretical molecular weight of 39.06 kDa, which falls within the optimal range for selecting promising vaccine targets [80]. An isoelectric point (pI) of 8.87 was predicted, indicating a basic character of the protein. Additionally, both the instability index (26.74) and the aliphatic index (84.15) were within acceptable ranges, suggesting that the protein is stable in vitro, even at elevated temperatures. This implies that TcGAPDH may retain its structural integrity during purification processes, including those involving heat treatment. The GRAVY score of –0.147 indicates a hydrophilic nature, suggesting that the protein can interact with aqueous environments, which favours its solubility and further supports its potential use as a vaccine candidate [81–84]. Regarding the estimated half-life, which were > 30, > 20, and > 10 h in mammals, yeast, and bacteria, respectively. These results suggest that TcGAPDH exhibits stability across different biological systems, providing an adequate window to support the induction of an effective immune response after vaccine administration [85]. The antigenicity analysis also revealed that TcGAPDH possesses favourable immunogenic properties, suggesting its potential to be recognised by the immune system and elicit an immune response. Moreover, the absence of predicted allergenicity represents a positive safety feature, as it minimises the risk of adverse allergic reactions [81, 86, 87].

The T and B cell epitope prediction, as well as the immune response simulation induced by TcGAPDH, demonstrated a complex and favourable immunological profile, with significant implications for the development of vaccine candidates against *T. cruzi*. Seven epitopes were identified as being recognised by MHC class I molecules, primarily associated with HLA alleles that are predominant in the Latin American population. These epitopes also contain predicted proteasomal cleavage sites, a feature that suggests differential processing at the proteasome level, which is crucial for the efficient generation of peptides and their subsequent presentation by the MHC-I pathway [53]. Additionally, five epitopes recognised by MHC class II molecules and six linear B cell epitopes were identified, maintaining the association with HLA alleles prevalent in the Latin American population. These findings demonstrate possible immune activation of the three key components of the adaptive immune response: the cytotoxic response mediated by CD8⁺ T lymphocytes, the CD4⁺ T cell-dependent helper response, and the humoral response mediated by B lymphocytes [88–90].

The activation of cytotoxic CD8⁺ T lymphocytes represent a critical step in eliminating *T. cruzi*-infected cells, constituting the most direct effector mechanism against the intracellular form of the parasite. Conversely, CD4⁺ T lymphocytes are characterized by the expression of surface molecules and the secretion of cytokines that modulate the activity of other immune cells, thus coordinating a sustained and effective immune response. Although B-cell activation leads to the production and secretion of antibodies with neutralizing and opsonizing capacities against the parasite, the humoral response by itself is insufficient for adequate clearance. Nevertheless, their importance lies in the establishment of *T. cruzi*-specific memory T cell populations, which are crucial for long-term infection control [91, 92].

A significant increase in IgM characterised the immune profile generated through the immune response simulation of TcGAPDH and IgG antibody levels, accompanied by elevated levels of key cytokines, including IFN-γ, IL-2, and IL-12. This immune response, characterized by a Th1-type profile, represents a central mechanism in controlling infection, due to the intracellular nature of the parasite. IL-12 plays a crucial role in promoting the differentiation and clonal expansion of Th1 CD4⁺ T helper cells and in inducing IFN-γ production by both Th1 CD4⁺ and CD8⁺ T cells, as well as by NK cells. IFN-γ enhances the activation of macrophage effector mechanisms and the generation of reactive oxygen species and reactive nitrogen species (ROS and RNOS), which are essential for the elimination of both amastigotes and phagocytosed trypomastigotes [15, 92]. IL-2 also plays a relevant role in the persistence of activated T lymphocytes and in the generation and maintenance of T cell memory, which ensures the long-term persistence of the adaptive immune response [91].

Our reverse vaccinology approach aligns with successful strategies employed in related kinetoplastids, where conserved metabolic and structural proteins have shown promise as vaccine candidates. In *Leishmania*, proteomic analyses of attenuated vaccine strains have identified GAPDH among upregulated Th1-stimulatory proteins with immunogenic potential [93], while metabolic enzymes such as pyruvate kinase have demonstrated protective efficacy when formulated with appropriate adjuvants, inducing IFN-γ production and Th1-biased immune responses in murine models [94]. Similarly, immunoinformatic approaches in *T. brucei* have successfully identified conserved antigenic proteins through computational epitope prediction, generating multi-epitope vaccine candidates predicted to elicit strong cellular and humoral responses [95]. Importantly, the Th1 cytokines profile predicted by our simulations is consistent with experimental evidence demonstrating that CD8 + T cell-mediated IFN-γ production is critical for *T. cruzi* control, and that multifunctional T cells producing IFN-γ, IL-2, and TNF-α represent established correlates of protection in both murine models and human Chagas disease [96–98].

The establishment of adaptive immunity mainly depends on the prior activation of innate immunity, which enables rapid recognition and response to the presence of antigens. In this context, TLRs represent one of the primary mechanisms through which the immune system detects pathogens and induce signalling pathways that lead to the production of proinflammatory cytokines and interferons, as well as the recruitment and activation of various effector cells [99]. In this study, molecular docking simulations revealed that TcGAPDH interacts favourably with the TLR-2 and TLR-4 receptors, exhibiting negative docking scores of –276.5 and –370.0, respectively. Subsequent analysis using PDBsum characterized the nature of the intermolecular interactions between TcGAPDH and both receptors. The results revealed multiple stabilizing interactions, including hydrogen bonds, salt bridges, and non-bonded contacts, with 20 TcGAPDH residues interacting with TLR-2 and 39 with TLR-4. The diversity of these interactions suggests a robust and multivalent binding interface, where each type of interaction contributes synergistically to complex stability. In this respect, hydrogen bonds provide directionality and interaction specificity, key features in molecular recognition. Salt bridges reinforce the ligand-receptor complex through electrostatic interactions, while non-bonded contacts promote molecular packing and surface complementarity, further contributing to the stabilization of the complex [100–103].

The interaction with TLR-2 and TLR-4 is particularly relevant in the context of *T. cruzi* infection, as both receptors play essential roles in the early recognition of the parasite and the activation of innate immune responses. TLR-2 has been associated with the recognition of GPI anchors derived from *T. cruzi* mucin-like glycoproteins (GPI-mucins) and is also involved in activating the small GTPase Rab-5, which induces parasite internalization by macrophages. TLR-4 has been associated with the recognition of glycoinositolphospholipids (GIPLs) present on the surface of the trypomastigote form. This recognition has significant implications for the activation of mitogen-activated protein kinase (MAPK) and nuclear factor kappa B (NF-κB) signalling pathways, consequently stimulating the production of RNOS and proinflammatory cytokines such as IL-12 and TNF-α. These molecules, in conjunction with previously identified cytokines such as IL-12 and IFN-γ, promote a proinflammatory microenvironment that favours Th1-type immune polarisation [15, 91, 92].

Molecular dynamics simulations revealed that the TcGAPDH–TLR2 and TcGAPDH–TLR4 complexes exhibited consistent structural behaviour throughout the simulation period, maintaining both stable intermolecular interactions and binding affinities. This temporal stability is indicative of consistent and functionally relevant interactions between TcGAPDH and the TLR-2 and TLR-4 receptors. Energy decomposition analysis further revealed the molecular basis of these interactions, demonstrating that electrostatic forces dominate the binding energetics in both complexes, accounting for approximately 90% of the total non-bonded energy, with Van der Waals forces contributing the remaining 10%. Notably, the TcGAPDH-TLR2 complex exhibited moderate electrostatic and Van der Waals contributions compared to the SSL3 reference, while the TcGAPDH-TLR4 complex showed energetic profiles remarkably similar to the MD-2-TLR4 reference system. This electrostatic dominance is characteristic of pathogen-associated molecular pattern (PAMP) recognition by Toll-like receptors, where charged and polar residues at the binding interface drive initial recognition and complex stabilization [104, 105]. The constant structural variation of both complexes are essential for effective recognition by the innate immune system, which enables the activation of coordinated adaptive immune responses. Additionally, it is a necessary condition for proper signal transduction through TLRs and the subsequent production of proinflammatory cytokines [106, 107].

An important consideration emerging from this analysis is the 53.29% sequence identity between TcGAPDH and human GAPDH, which requires careful discussion regarding potential autoimmunity risks. While this level of homology is substantial, several factors must be considered when evaluating its implications for vaccine development. Overall protein sequence identity is an imperfect predictor of autoimmunity risk, as cross-reactivity depends primarily on the specific epitopes presented to the immune system rather than global sequence conservation. GAPDH proteins characteristically show high conservation in catalytic core domains while exhibiting substantial divergence in surface loops, terminal regions, and non-catalytic domains [108]. Our epitope predictions identified immunogenic regions distributed across the protein sequence, some of which may map to these divergent regions. Notably, successful vaccine candidates have been developed from pathogen proteins despite sharing substantial sequence homology with human orthologs, for example, *Plasmodium falciparum* GAPDH, which shares 63% sequence identity with human GAPDH[109, 110].These precedents indicate that overall sequence identity, while warranting careful attention, does not automatically preclude development if epitope-level analysis and experimental validation demonstrate acceptable cross-reactivity profiles. However, it is essential to acknowledge that this study presents in silico predictions that require experimental validation to confirm biological relevance. Multiple strategies should be implemented in subsequent development stages to assess and mitigate autoimmunity concerns, including: epitope-level homology screening to identify and exclude highly conserved peptides, peptide synthesis and competitive MHC binding assays for predicted epitopes, peptide-based cross-reactivity assays with human GAPDH, functional TLR activation assays using recombinant proteins, comprehensive immunological profiling in appropriate animal models to detect potential autoimmune responses, and validation of immune response predictions through preclinical immunization studies in murine models with *T. cruzi* experimental challenge. If necessary, reformulation as a multi-epitope vaccine containing only divergent peptide sequences could be considered. This integrated experimental pipeline will be essential to translate these in silico predictions into viable vaccine candidates and to ensure that the sequence homology with human GAPDH does not pose unacceptable cross-reactivity risks.

Meanwhile, the convergence of innate (via TLRs) and adaptive (via antigen presentation) signalling pathways synergistically enhances the anti-*T. cruzi* immune response, establishing a critical link between innate and adaptive immunity that is fundamental for the effective control of parasitic infection. The identification of multiple epitopes, combined with its ability to induce a Th1 immune response, suggests that TcGAPDH is an up-and-coming vaccine candidate. Its specific association with HLA alleles predominant in populations across the Americas, along with its capacity to simultaneously activate both cellular and humoral immune responses, constitutes a major advantage for evaluating it as a vaccine development against Chagas disease.

## Conclusion

The absence of an effective and safe treatment for Chagas disease highlights the urgent need to explore new therapeutic and preventive strategies, including vaccine development. The results obtained from the in silico analysis provide evidence that *T. cruzi* TcGAPDH exhibits immunogenic and physicochemical properties that position it as a promising vaccine candidate. Experimental validation of these findings will be crucial to advancing toward novel strategies that contribute to the control and eventual eradication of the disease.

## Supplementary Information

Below is the link to the electronic supplementary material.

Supplementary Material 1

## Data Availability

No datasets were generated or analysed during the current study.
