# Supplementary material for: In Silico Design and Evaluation of TcGAPDH as a Vaccine Against Chagas Disease: A Reverse Vaccinology Approach
Source: Acta Parasitol. 2026 Feb 16;71(1):40. doi: 10.1007/s11686-026-01221-4 (PMC12909448; doi:10.1007/s11686-026-01221-4)
Supplement: Supplementary file 1 — Supplementary Material 1 [file 11686_2026_1221_MOESM1_ESM.docx]

**Supplementary material**


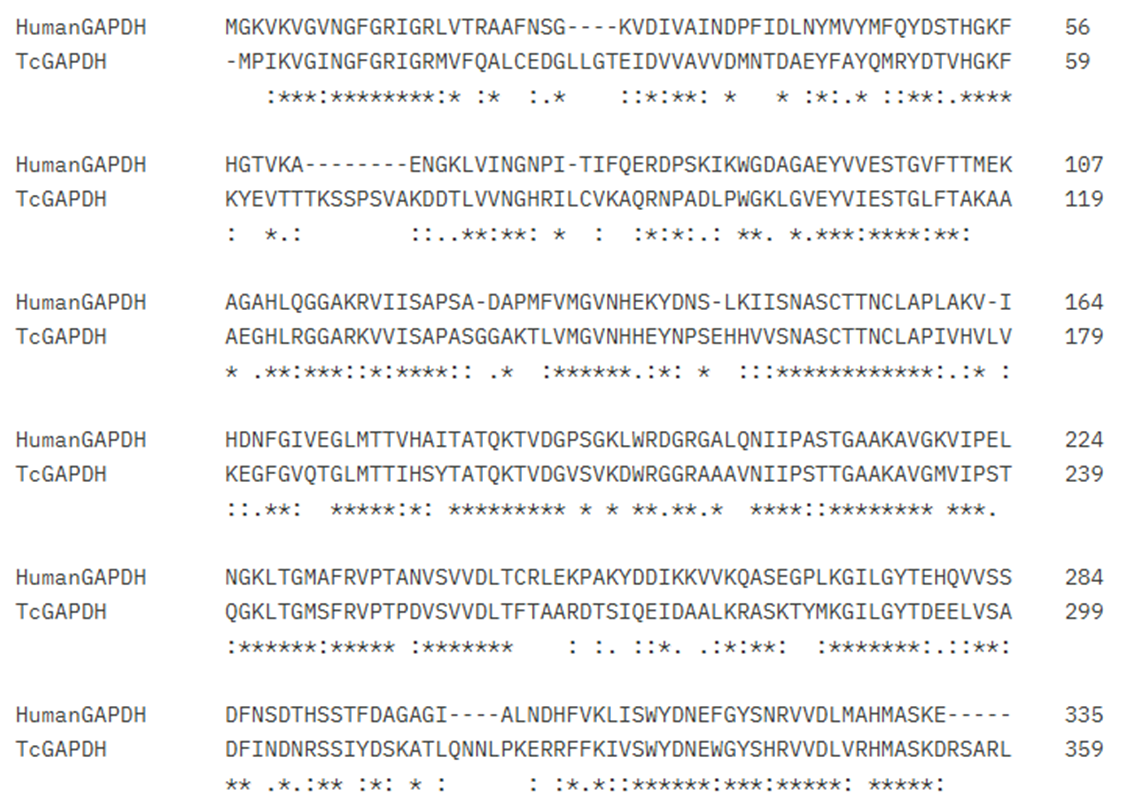


1. Aligment analysis of the amino acid sequences of the TcGAPDH (BCY84_01123_t1) and human GAPDH (P04406). Asterisks indicated the conserved amino acids in both sequences; conservative and semiconservative amino acids are labeled with two and one point, respectivaly; and dashed lines indicated gaps introduced between the sequences.
2. MolProbity assessment values for the refined protein structures.

| **Parameter** | **TcGAPDH** | **TLR-2** | **TLR-4** | **MD2** | **SSLP3** | **Ideal Values** |
| --- | --- | --- | --- | --- | --- | --- |
| MolProbity score | 1.62 | 2.21 | 2.15 | 2.16 | 1.25 | <1.5 |
| Clashscore | 1.46 | 3.64 | 2.2 | 3.88 | 0.94 | <5 |
| Ramachandran favored | 95.24% | 88.67 | 92.99 | 96.48 | 95.74 | >98% |
| Ramachandran outliers | 0.28% | 1.10 | 0.17 | 0.70 | 0.00 | <0.2% |


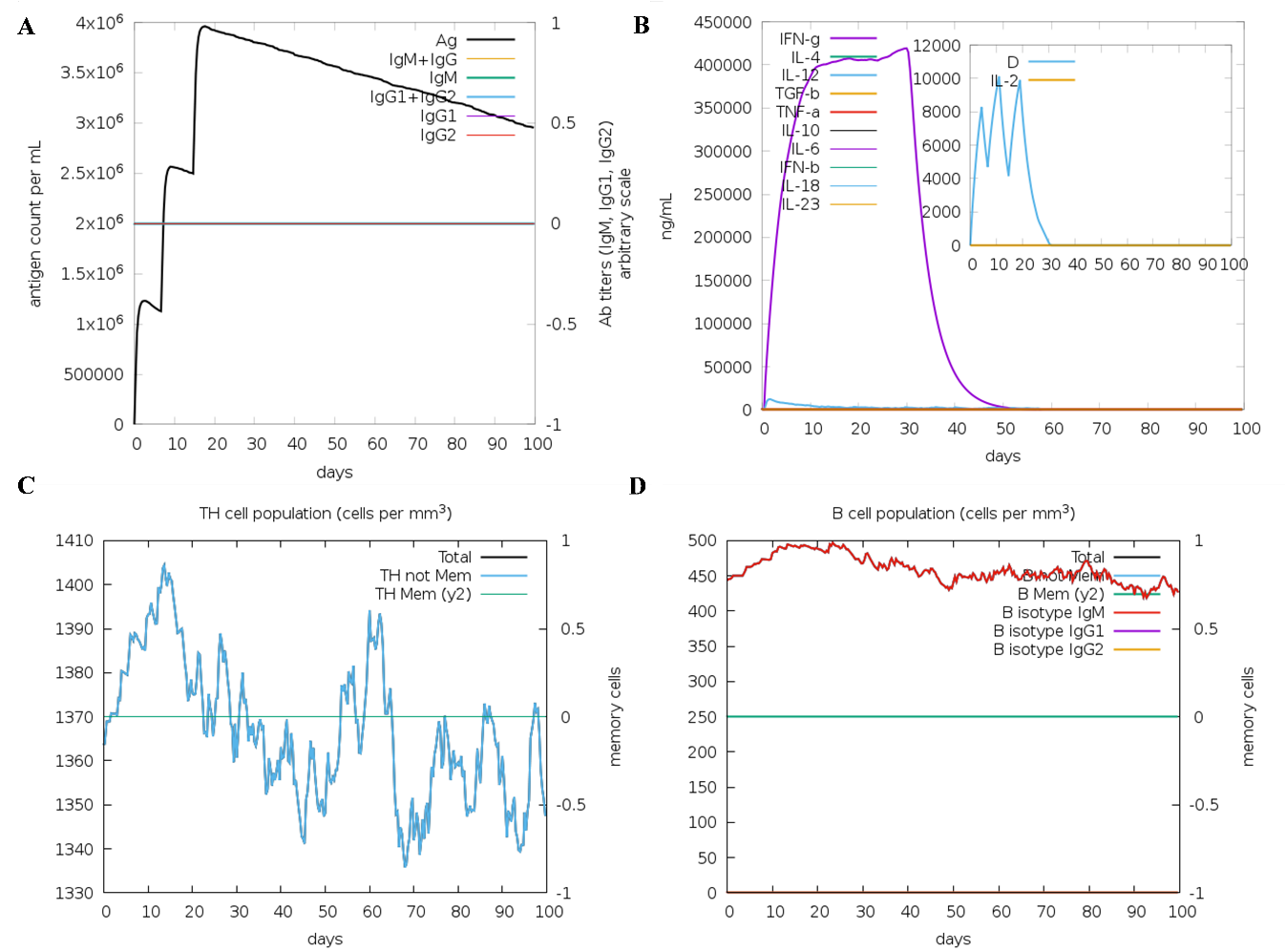


1. Negative control immune response simulation with a short non-immunogenic peptide (GGGGGG).A) Absense of predictive antibody production, all antibody isotypes remaining at zero levels throughout the simulation period. B) Cytokine profile demonstrating low baseline levels without significant immune activation. The inset shows detailed temporal dynamics of selected cytokines. C) TH cell population remains stable without expansion or memory cell (y2) differentiation. D) B cell population maintains basal levels without expansion, isotype switching, or memory B cell formation.
